# Scalar wave diffraction by an open-ended sphere-conical cavity: the Abel integral transform in the Dirichlet and Neumann problems

**DOI:** 10.1098/rsta.2024.0338

**Published:** 2025-08-14

**Authors:** Dozyslav Kuryliak, Victor Lysechko

**Affiliations:** ^1^ Department of the Theory of Wave Processes and Optical Systems of Diagnostics, Karpenko Physico-Mechanical Institute of the NAS of Ukraine, Lviv, Ukraine

**Keywords:** truncated cone, sphere-conical cavity, analytical regularization, convolution-type operator

## Abstract

Two scalar wave diffraction problems for an open-ended sphere-conical cavity formed by a semi-infinite truncated cone with an internal termination in the form of the spherical cap in one of the conical regions are considered in the case of an axial excitation by a plane wave. The problems are formulated in terms of mixed boundary value ones with respect to the scalar potentials for the Helmholtz equation with Dirichlet or Neumann boundary conditions. Our technique is based on the mode matching, which is applied to reduce the problems to the infinite system of linear algebraic equations (ISLAEs) of the second kind by the method of analytical regularization. This includes the Abel integral transformation of the Legendre series equation to the Dirichlet one to justify the unique transition to ISLAE, separating the singular operators from them and deriving their inverse ones. To extend the applicability of our technique, two types of the regularization procedures are applied for the solutions, and the general scheme for designing the family of regularizing operators is proposed. The analytical solutions of the problems are obtained for small size of the cavity aperture. Based on this, the new approximate formulas are obtained to determine the cavity resonance frequency perturbations. Depending on the geometry parameters and the physical interpretation of the potentials, the scattering characteristics of probes, reflectors, resonators and subsurface defects are analysed numerically for two limiting cases of the physical properties of the scatterer’s surfaces.

This article is part of the theme issue ‘Analytically grounded full-wave methods for advances in computational electromagnetics’.

## 1. Introduction

We study scalar wave diffraction from an open-ended sphere-conical cavity that is formed by the junction of two canonical geometries: the semi-infinite truncated circular cone, which is obtained by truncations of its apex and an internal spherical cap termination, which closes one of the hollow conical sectors. The conical wall of the cavity is assumed to be infinitely thin. Since the scatterer mentioned above consists of fragments of canonical geometry that can be described in a unit spherical coordinate system, this article aims to develop an accurate technique for studying the corresponding wave diffraction problem. The interest in this geometry lies in its potential wide application for modelling various problems of applied physics and engineering. This cavity can be considered as the working element of waveguide probes for microwave diagnostics [1]. If the spherical cap is located near an open end of the cavity, it can be used as a reference model for design of the tripod-supported concave spherical reflectors and antennas [2,3]. If the cavity degenerates into the hemi-spherical one, it becomes the model of the under surface defect [4] and has the potential to be the panel’s cell of the metamaterials for modelling absorbing surfaces [5–7].

To obtain the solution, we formulate a mixed boundary-value problem with respect to the scalar potential for the Helmholtz equation. Our theory may correspond to acoustic or electromagnetic problems, including static ones. For further convenience, we analyse the velocity field potential for the acoustic waves. In order to estimate the influence of the physical properties of the scattered surfaces on the diffracted fields, we consider the Dirichlet and Neumann problems.

The reference solutions of wave diffraction from the open-ended cavities formed by the fragments of the canonical geometries are mostly studied in Cartesian and cylindrical coordinates for acoustic and electromagnetic waves. The wave diffraction problems from parallel plate [8,9] and cylindrical [10–12] open-ended cavities were analysed using the Fourier transformation and the Wiener–Hopf technique. Obtaining these solutions required a modification of the canonical Wiener–Hopf method to ensure the fulfilment of the boundary conditions at the termination. Therefore, the corresponding solutions create the theoretical basis to study the scattering properties for more complicated structures. The Wiener–Hopf technique and the Kontorovich–Lebedev integral transformation were applied to solve wave diffraction problems from open and closed cones [13–15]. This technique was also used to obtain a high-frequency asymptotic solution to the wave diffraction problem from a spherical cap [16,17]. Due to the number of restrictions for applying the Wiener–Hopf technique in a spherical coordinate system and in order to extend the geometries that allow for obtaining the rigorous analysis the analytical regularization methods were developed. These methods are based on the mode matching and use the analytical inversion of the singular parts of the corresponding equations. An analytical regularization technique focused on solving the problems of wave diffraction from open spherical resonators was developed in [18,19]. The Abel integral transformation and Fourier series theory were used to invert the singular part of these problems. The analytical regularization technique for solving the wave diffraction problems from the finite and truncated hollow cones was early developed by the authors in [20–22]. In [23,24], we modified this technique to study the wave diffraction problems from the open-ended sphere-conical cavities in the electromagnetic case and analysed the axially symmetric excitation by the vertical electric dipole.

In this paper, we obtain the accurate solutions of new mixed boundary value problems of wave diffraction by the soft and rigid sphere-conical cavities, using our methodology developed early in [20–24]. Our analysis is based on the mode matching for reducing the problems to the functional series equations. Due to the fields’ singularities at the conical edge, these equations are represented in the form of a limiting transition to infinite numbers of the accounted eigenmodes. We apply the Abel integral transformation to reduce them to the Dirichlet series. This is a new step in our theory that allows us to justify the transition from the series equations to an infinite system of linear algebraic equations (ISLAEs) and ensure the correct selection of their singular parts, which are the convolution-type operators. One of the key steps in our analysis is the derivation of the inverse operators from the singular ones using the factorization procedure of the problems’ kernel functions. Couples of the convolution-type operators and the corresponding inverse ones are used to reduce the problems to ISLAE of the second kind by the method of analytical regularization. We apply two types of regularization procedures, namely, left- and right-hand regularizations and designing of the family of the regularizing operators for our problems. These new regularizing operators extend the applicability of the proposed technique as well as simplify its application. The analytical solutions of our problems are obtained for the small-size cavity aperture. Based on this, the new approximate formulas are derived to determine the cavity resonance frequency perturbations. Depending on geometry, the resonant scattering characteristics of probes, reflectors, open resonators and hidden subsurface defects are analysed numerically for two limiting cases of the physical properties of the scatterers.

It is worth noting that the waves scattering from the finite hollow and closed cones using different methods has been the subject of many studies in the literature [25–30]. In the context of potential theory, the hollow truncated cone and finite closed one were examined accurately in [31,32] using the Mellin integral transformation and Wiener–Hopf technique.

## 2. Formulation of the problem

**Figure 1.**
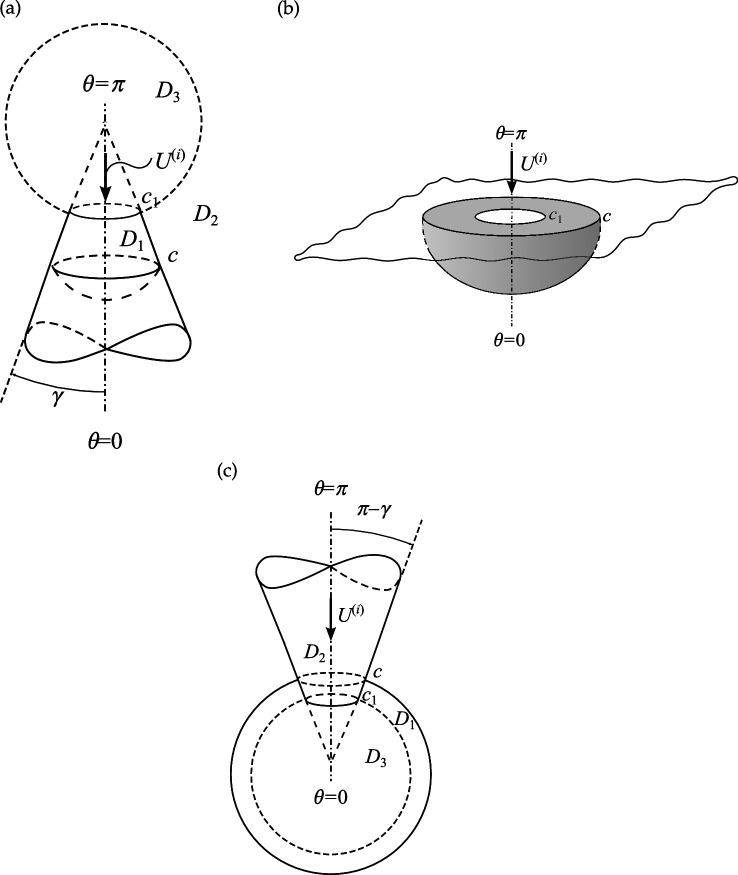
Geometry of the open-ended sphere-conical cavities: (a) an open-ended sphere-conical resonator, γ<π/2; (b) an open hemi-spherical cavity under the plane; (c) an open spherical resonator conjoined with the conical horn.


Let us consider a semi-infinite truncated circular cone with an infinitely thin wall. An internal perfectly conducting spherical cap termination in one of two conical sectors forms an open sphere-conical resonator (figure 1). In the spherical coordinates (r,θ,φ), this structure can be expressed as

$$Q=\left\{(r,\theta ,\varphi )|\;r\in \left\{\begin{array}{l} \left(c_{1},\infty \right),\theta =\gamma +0\\ \left(c_{1},c\right),\theta =\gamma -0 \end{array}\right\},\theta =\gamma \right\}\underset{\varphi \in [0,2\pi )}{⋃}\{(r,\theta ,\varphi )|r=c,\theta \in [0,\gamma ]\},$$

where γ is the spherical aperture angle; $c_{1}$ is the radial coordinates of the rib of the hole; c is the radius of the spherical diaphragm, $c>c_{1}$. Let us consider the cone *Q* in a homogeneous isotropic medium. This cone is irradiated by a plane acoustic wave, which propagates along the positive direction of the z-axis and is defined by the velocity potential of unit amplitude as


(2.1) $$\begin{array}{lcr} & U_{0}(r,\theta )=e^{ikr\;\cos\;\theta }. & \end{array}$$

Here, k=ω/υ=2π/λ is the wavenumber, ω is the angular frequency, υ and λ are the velocity and wavelength of the sound wave, respectively. Time factor $e^{-i\omega t}$ is suppressed throughout this paper. The problem is to determine the fields formed by the plane wave (2.1) in the partial domains formed by the scatterer *Q*


(2.2) $$\begin{array}{lcr} & \begin{array}{rl} D_{1} & :\left\{r\in (c_{1},c);\theta \in [0,\gamma )\right\},\\ D_{2} & :\left\{r\in (c_{1},\infty );\theta \in (\gamma ,\pi ]\right\},\\ D_{3} & :\{r\in (0,c_{1});\theta \in [0,\pi ]\}. \end{array} & \end{array}$$

It is clear that φ∈[0,2π) for each region. Taking into account the fact that $D_{2}$ includes the infinity area let us represent the total field in $D_{2}$ as the sum of an initial one $U^{\left(i\right)}(r,\theta )$, which is the field of the plane wave (2.1) in a semi-infinite cone {0<r<∞,γ<θ≤π} obtained earlier in [22] and the unknown pertubation/diffracted field U(r,θ) caused by the edge and radiated to infinity:


(2.3) $$U^{\left(i\right)}(\rho ,\theta )=\sqrt{\frac{1}{\rho }}\sum_{k=1}^{\infty }a_{\mu_{k}}^{\left(0\right)}P_{\mu_{k}-1/2}(-\cos\theta )I_{\mu_{k}}(\rho ),$$

where


$$a_{\mu_{k}}^{\left(0\right)}=\left\{ \begin{array}{l} \frac{\sqrt{2\pi^{3}}\;\mu_{k}\;P_{\mu_{k}-1/2}(\cos\gamma )}{\cos(\pi \mu_{k})\;\partial_{\mu }P_{\mu_{k}-1/2}(-\cos\gamma )},\;k\geq 1,\;\text{D-problem}\\ \left\{ \begin{array}{l} \sqrt{\frac{\pi }{2}}\left[1+\tan^{2}\left(\frac{\gamma }{2}\right)\right],\;\;k=1,\\ -\frac{\sqrt{2\pi^{3}}\;\mu_{k}\;P_{\mu_{k}-1/2}^{1}(\cos\gamma )}{\cos(\pi \mu_{k})\;\partial_{\mu }P_{\mu_{k}-1/2}^{1}(-\cos\gamma )},\;k>1, \end{array}\right.\;\text{N-problem} \end{array}\right.$$

$P_{\eta -1/2}(\cdot )$ is the Legendre function; $I_{\eta }(\cdot )$ is the modified Bessel function; ρ=sr, s=−ik. Now, we define the total field of our problem as


(2.4) $$\begin{array}{lcr} & \begin{array}{r} U^{\left(t\right)}(r,\theta )=\left\{ \begin{array}{cc} U(r,\theta ), & (r,\theta )\in D_{1},D_{3};\\ U^{\left(i\right)}(r,\theta )+U(r,\theta ), & (r,\theta )\in D_{2}. \end{array}\right. \end{array} & \end{array}$$

To determine the diffracted field let us formulate a mixed boundary value problem for the Helmholtz equation


(2.5) $$\begin{array}{lcr} & \Delta U(r,\theta )+k^{2}U(r,\theta )=0, & \end{array}$$

where Δ is the Laplace operator,


$$\begin{array}{c} \Delta =\partial_{rr}+2r^{-1}\partial_{r}+(r^{2}\sin\;\theta {)}^{-1}\partial_{\theta }\left(\sin\;\theta \;\partial_{\theta }\right). \end{array}$$

The unknown field U(r,θ) satisfies the Dirichlet/Soft (D/S) or Neumann/Rigid (N/R) boundary conditions on the scattering surfaces in the form


$$\begin{array}{lcr} & {U^{\left(t\right)}(r,\theta )|}_{(r,\theta )\in Q}=0,\text{ D-problem; } & \text{(2.6a)}\\ & \left\{ \begin{array}{l} \partial_{\theta }U^{\left(t\right)}(r,\theta )=0\text{ if }r\in \left\{\begin{array}{c} \left(c_{1},\infty \right),\theta =\gamma +0\\ \left(c_{1},c\right),\theta =\gamma -0 \end{array}\right\},\text{ N-problem. }\\ \partial_{r}U^{\left(t\right)}(r,\theta )=0\text{ if }r=c,\theta \in [0,\gamma ] \end{array}\right. & \text{(2.6b)} \end{array}$$

We search the solution of the mixed boundary value problems (2.5), (2.6a) and (2.5), (2.6b) in a class of functions that satisfy the radiation condition for the diffracted field


(2.7) $$\begin{array}{lcr} & \lim_{r\to \infty }\;r[\partial_{r}U(r,\theta )-ikU(r,\theta )]=0 & \end{array}$$

as well as the condition of finiteness of energy in any bounded volume. In our case, the second condition is reduced to the fulfilment of the Meixner one at the edge of Q as


(2.8) $$\begin{array}{lcr} & \mathrm{grad}U(r,\theta )=O({\overset{⌢}{\rho }}^{-1/2}), & \end{array}$$

where $\overset{⌢}{\rho }$ is the distance to the edge in the local coordinate system. Due to these two additional conditions, our diffraction problem is well posed.

## 3. Field representations

In order to satisfy the Helmholtz equation (2.5) for each region (2.2) together with appropriate boundary conditions (2.6a) and (2.6b) on Q, we seek the scattered field for D- and N-problems in the form as


(3.1) $$\begin{array}{lcr} & \begin{array}{r} U(\rho ,\theta )=\frac{1}{\sqrt{\rho }}\left\{ \begin{array}{cc} \sum_{p=1}^{\infty }P_{\nu_{p}-1/2}(\cos\;\theta )\left[y_{p}^{\left(1\right)}\frac{K_{\nu_{p}}(\rho )}{K_{\nu_{p}}(\rho_{1})}+y_{p}^{\left(1;1\right)}\frac{I_{\nu_{p}}(\rho )}{I_{\nu_{p}}(\rho_{c})}\right], & (r,\theta )\in D_{1},\\ \sum_{k=1}^{\infty }y_{k}^{\left(2\right)}P_{\mu_{k}-1/2}(-\cos\;\theta )\frac{K_{\mu_{k}}(\rho )}{K_{\mu_{k}}(\rho_{1})}, & (r,\theta )\in D_{2},\\ \Phi (\rho )+\sum_{n=1}^{\infty }{\bar{x}}_{n}^{\left(1\right)}P_{z_{n}-1/2}(\cos\;\theta )\frac{I_{z_{n}}(\rho )}{I_{z_{n}}(\rho_{1})}, & (r,\theta )\in D_{3}. \end{array}\right. \end{array} & \end{array}$$

Here, $y_{p}^{\left(1\right)}$, $y_{p}^{\left(1;1\right)}$, $y_{k}^{\left(2\right)}$, ${\bar{x}}_{n}^{\left(1\right)}$ are unknown expansion coefficients to be determined; $K_{\eta }(\cdot )$ is the Macdonald function; $\rho_{1}=sc_{1}$, $\rho_{c}=sc$;


(3.2a)zn=n−1/2 with Φ(ρ)≡0 for D-problem (3.2b)andzn=n+1/2withΦ(ρ)=x¯0(1)I1/2(ρ)/I1/2(ρ1),wherex¯0(1)isunknownforN-problem;

$\nu_{p},\;\mu_{k}$ are real positive roots of transcendental equations [33,34]


$$\begin{array}{lrlr} & & {P_{\eta -1/2}(\cos\;\gamma )|}_{\eta =\nu_{p}}=0,{P_{\eta -1/2}(-\cos\;\gamma )|}_{\eta =\mu_{k}}=0,\;\text{ D-problem, } & \text{(3.3a)}\\ & & {P_{\eta -1/2}^{1}(\cos\;\gamma )|}_{\eta =\nu_{p}}=0,{P_{\eta -1/2}^{1}(-\cos\;\gamma )|}_{\eta =\mu_{k}}=0,\;\text{ N-problem, } & \text{(3.3b)} \end{array}$$

where $P_{\eta -1/2}^{1}(\cdot )$ is the associated Legendre function defined [35] as


$$\begin{array}{c} P_{\eta -1/2}^{1}(\pm \cos\;\gamma )=\pm \partial_{\gamma }P_{\eta -1/2}(\pm \cos\;\gamma ). \end{array}$$

Note that, the first positive roots of the transcendental (3.3b) $\nu_{1}=\mu_{1}=1/2$ are not dependent on the opening angle γ. Taking this into account, as well as the equality $P_{0}(\cos\;\theta )=1$, which is correct for any 0≤θ≤π, we find that the field representation (3.1) contains the terms that do not depend on the azimuthal coordinate θ in the region $D_{3}$ for D-problem and for each region $D_{1}$, $D_{2}$, $D_{3}$ for *N* one. These terms in (3.1) consider the excitation of the piston waves in the above-mentioned regions. Using the boundary condition (2.6) and representation (3.1), we have arrived at the equation as


(3.4) $$\begin{array}{lcr} & y_{p}^{\left(1;1\right)}=y_{p}^{\left(1\right)}\Upsilon_{\nu_{p}}(\omega )\frac{K_{\nu_{p}}(\rho_{c})}{K_{\nu_{p}}(\rho_{1})}, & \end{array}$$

where


(3.5) $$\Upsilon_{\nu_{p}}(\omega )=\left\{ \begin{array}{ll} -1, & \text{ D-problem, }\\ -\frac{1-2\rho_{c}K_{\nu_{p}}^{\prime }\left(\rho_{c}\right)/K_{\nu_{p}}\left(\rho_{c}\right)}{1-2\rho_{c}I_{\nu_{p}}^{\prime }\left(\rho_{c}\right)/I_{\nu_{p}}\left(\rho_{c}\right)}, & \text{ N-problem. } \end{array}\right.$$

Here, p=1,2,3,…; the prime indicates the derivation of the modified Bessel and Macdonald functions with respect to the argument.

It should be noted that according to the definition (2.4) and the expressions (2.3), (3.3) and (3.1), the boundary conditions (2.6a) and (2.6b) are complete to be enforced in the subregions $D_{1}$, $D_{2}$ and $D_{3}$. The representation (3.1) provides the radiation condition at infinity (2.7) and, as follows from (3.2), is limited in the beginning of the spherical coordinates. To satisfy the Meixner condition (2.8) at the aperture’s edge, we search the unknown expansion coefficients in a class of sequences $\varsigma_{n}=O(n^{-1})$ for n→∞, where $\varsigma_{n}={\bar{x}}_{n}^{\left(1\right)}(y_{n}^{\left(1\right)},y_{n}^{\left(2\right)})$.

## 4. Matching series equations

To find the unknown expansion coefficients in (3.1), we use the mode matching of the total field (2.4) and its normal derivative on the virtual spherical surface of the radius $r=c_{1}$ containing the circular aperture of a truncated cone. This leads to the functional (series) equations with respect to unknown expansion coefficients with Legendre function kernel, defined on θ∈[0,π]. The behaviour of the gradU(r,θ) in the vicinity of the cone’s edge has a singularity of the form (2.8). Keeping this in mind and using the correlations (3.4) and (3.5), we present these equations by way of limiting passing for both D- and N-problems as


Φ(ρ1)+limN→∞∑n=1Nx¯n(1)Pzn−1/2(cos⁡θ)(4.1a)={limP→∞∑p=1Pyp(1)Pνp−1/2(cos⁡θ)[1+Υνp(ω)Kνp(ρc)Iνp(ρc)Iνp(ρ1)Kνp(ρ1)],θ∈[0,γ),ρ1U(i)(ρ1,θ)+limK→∞∑k=1Kyk(2)Pμk−1/2(−cos⁡θ),θ∈(γ,π],

Φ′(ρ1)+limN→∞∑n=1Nx¯n(1)Pzn−1/2(cos⁡θ)Izn′(ρ1)Izn(ρ1)(4.1b)={limP→∞∑p=1Pyp(1)Pνp−1/2(cos⁡θ)[Kνp′(ρ1)Kνp(ρ1)+Υνp(ω)Kνp(ρc)Iνp(ρc)Iνp′(ρ1)Kνp(ρ1)],θ∈[0,γ),[ρ1U(i)(ρ1,θ)]ρ′+limK→∞∑k=1Kyk(2)Kμk′(ρ1)Kμk(ρ1)Pμk−1/2(−cos⁡θ),θ∈(γ,π],

where the prime denotes derivative with respect to the argument. The main reason of this limitation is to provide the correct transition from (4.1a) and (4.1b) to ISLAE (P,K,N→∞), the solution of which satisfies the Meixner condition at the conical edge.

Taking into account the asymptotic behaviour of the unknown coefficients (3.1) represented in §3, it is found that the series in equation (4.1a) are uniformly and absolutely convergent in the corresponding areas of the angle θ because the terms of the series in the equation mentioned above decrease as $\eta^{-3/2}$ if η→∞, where η=n(p,k). The terms of the series in (4.1b) asymptotically decrease as $\eta^{-1/2}$ for η→∞. Therefore, we suppose that these series are conditionally convergent, and as follows from this, their sums depend on the order of summation. Then, our problem is to obtain the unique solution of the series equation (4.1), which fulfils the Meixner condition at the cone edge.

## 5. Transition to the ISLAE

By virtue of orthogonal properties of the Legendre functions $\{P_{\nu_{p}-1/2}(\cos\;\theta ){\}}_{p=1}^{\infty }$ and $\{P_{\mu_{k}-1/2}(-\cos\theta ){\}}_{k=1}^{\infty }$ in angles areas θ∈[0,γ) and θ∈(γ,π]*,* respectively, the following representation may be written


(5.1) $$\begin{array}{lcr} & P_{z_{n}-1/2}(\cos\;\theta )=q(z_{n},\gamma )\left\{ \begin{array}{cc} \lim_{P\to \infty }\;\sum_{p=1}^{P}\frac{\nu_{p}\alpha^{+}(\nu_{p},\gamma )}{\nu_{p}^{2}-z_{n}^{2}}P_{\nu_{p}-1/2}(\cos\;\theta ), & \theta \in [0,\gamma ),\\ \lim_{K\to \infty }\;\sum_{k=1}^{K}\frac{\mu_{k}\alpha^{-}(\mu_{k},\gamma )}{\mu_{k}^{2}-z_{n}^{2}}P_{\mu_{k}-1/2}(-\cos\;\theta ), & \theta \in (\gamma ,\pi ]. \end{array}\right. & \end{array}$$

Here, we employ the notation


$$\begin{array}{c} q(z_{n},\gamma )=\left\{ \begin{array}{c} P_{z_{n}-1/2}(\cos\;\gamma )\\ P_{z_{n}-1/2}^{1}(\cos\;\gamma ) \end{array}\right.,\;\alpha^{\pm }(\eta ,\gamma )=-2\left\{ \begin{array}{c} {\left[\partial_{\nu }P_{\nu -1/2}(\pm \cos\;\gamma )\right]}_{\nu =\eta }^{-1}\\ \pm {\left[\partial_{\nu }P_{\nu -1/2}^{1}(\pm \cos\;\gamma )\right]}_{\nu =\eta }^{-1} \end{array}\right., \end{array}$$

where the upper and underlines correspond to D- and N-problems, respectively; the upper signs and $\eta =\nu_{p}$ correspond to θ∈[0,γ) and the lower one and $\eta =\mu_{k}$ correspond to θ∈(γ,π]. The asymptotic decaying terms of the series (5.1) look like $\lambda^{-1}$ and $\lambda^{-2}$ if λ→∞ for D- and N-problems, where λ=p(k). Considering this, we find that the series (5.1) are absolutely convergent for N- and formal for D-problems. For the further operation of equality (5.1), let us formulate the theorem.

**Theorem 5.1.** *For any angles* θ *that belong to the interval* 0≤θ<γ *or* γ<θ≤π, *the series* (5.1) *is represented by the uniformly convergent integral as*


(5.2) $$\begin{array}{lcr} & J_{n}^{\pm }(\theta )=\frac{1}{2\pi i}\int_{C_{R}}\frac{tP_{t-1/2}(\pm \cos\;\theta )}{\left(t^{2}-z_{n}^{2}\right)P_{t-1/2}^{l}(\pm \cos\;\gamma )}dt, & \end{array}$$

*where* $C_{R}$ *is the circular integration path of the radius* R *in the complex plane* t*; the upper sign refers to* θ∈[0,γ) *and the lower sign refers to* θ∈(γ,π]*;* l∈{0,1}*,* l=0 *and* l=1 *correspond to D- and N-problems, respectively*.

*Proof*. Singularities of the integrand (5.2) associated with the simple poles at $t=\pm z_{n}$ and $t=\pm \eta_{p}$, where $\eta_{p}=\nu_{p}$ if θ∈[0,γ) and $\eta_{p}=\mu_{p}$ if θ∈(γ,π]*,* p=1,2,3,…. As far as the integrand (5.2) decays as t−εe−|θ−γ||Im(t)| if |t|→∞, where ε=1 for the soft cone and ε=2 for the rigid one, then according to the Jordan lemma, we find that $J_{n}^{\pm }(\theta )\to 0$ if R→∞ and $C_{R}$ does not cross the singularities of the integrand. Therefore, using the residues theorem, let us represent the integral (5.2) as


(5.3) $$\begin{array}{lcr} & \begin{array}{c} \lim_{K(P)\to \infty }\;\sum_{k=1}^{K(P)}\frac{\eta_{k}P_{\eta_{k}-1/2}(\pm \cos\;\theta )}{(\eta_{k}^{2}-z_{n}^{2}){\partial_{t}P_{t-1/2}^{l}(\pm \cos\;\gamma )|}_{t=\eta_{k}}}\\ -\lim_{K(P)\to \infty }\;\sum_{k=1}^{K(P)}\frac{\eta_{k}P_{-\eta_{k}-1/2}(\pm \cos\;\theta )}{(\eta_{k}^{2}-z_{n}^{2}){\partial_{t}P_{t-1/2}^{l}(\pm \cos\;\gamma )|}_{t=-\eta_{k}}}\\ +\frac{P_{z_{n}-1/2}(\pm \cos\;\theta )}{2P_{z_{n}-1/2}^{l}(\pm \cos\;\gamma )}+\frac{P_{-z_{n}-1/2}(\pm \cos\;\theta )}{2P_{-z_{n}-1/2}^{l}(\pm \cos\;\gamma )}=0. \end{array} & \end{array}$$

Taking into account equality $P_{\nu -1/2}^{l}(\cos\;\theta )=P_{-\nu -1/2}^{l}(\cos\;\theta )$, we find that


(5.4) $$\begin{array}{lcr} & P_{z_{n}-1/2}(\cos\;\theta )=-{\left(\pm \right)}^{l}2P_{z_{n}-1/2}^{l}(\cos\;\gamma )\;\lim_{K(P)\to \infty }\;\sum_{k=1}^{K(P)}\frac{\eta_{k}P_{\eta_{k}-1/2}(\pm \cos\;\theta )}{\left(\eta_{k}^{2}-z_{n}^{2})\partial_{t}P_{\eta_{k}-1/2}^{l}(\pm \cos\;\gamma \right)}, & \end{array}$$

where l=0 corresponds to D and l=1 to the N-case, respectively. The representation (5.4) directly leads to the expression (5.1), that proves the theorem.∎

**Corollary 1.** *This theorem shows the alternative derivation of the equality (**5.1**) and explains the uniform convergence of the series (**5.1**) to the Legendre functions in the sense of the uniform convergence of the integral (**5.2**)*.

Let us substitute a relationship (5.1) into the left-hand side of series equations (4.1a) and (4.1b) for each of regions. As a result, we arrive at the four homogeneous series equations that take the form of


(5.5) $$\begin{array}{lcr} & \lim_{\begin{array}{c} J\to \infty ,\\ N\to \infty \end{array}}\;\sum_{j=1}^{J}\Lambda_{j}(x_{1},\ldots ,x_{N};y_{j})P_{\eta_{j}-1/2}(\pm \cos\;\theta )=0 & \end{array}$$

if 0≤θ<γ and $\eta_{j}=\nu_{j}$ for the upper sign and γ<θ≤π and $\eta_{j}=\mu_{j}$ for the lower one; $\Lambda_{j}(x_{1},\ldots ,x_{N};y_{j})$ is the known linear form, which follows from equations (4.1a) and (4.1b); $y_{j}\in \{y_{p}^{\left(1\right)}\}\vee \{y_{k}^{\left(2\right)}\},\;x_{n}\in \{{\bar{x}}_{n}^{\left(1\right)}\}$. Taking into account the linear independence of the finite set of Legendre functions $P_{\eta_{j}-1/2}(\pm \cos\;\theta )$, j=1,2,…,J, we find that for any finite J equation (5.5) has only the trivial solution


(5.6) $$\begin{array}{lcr} & \Lambda_{j}(x_{1},\ldots ,x_{N};y_{j})=0. & \end{array}$$

Therefore, from equation (5.6) directly follows the finite system of linear algebraic equations that we represent as


(5.7a)∑n=1Nxn(1)νp2−zn2=yp(1)νpα+(νp,γ)[1+Υνp(ω)Kνp(ρc)Iνp(ρc)Iνp(ρ1)Kνp(ρ1)],p=(1),2,3,…,P,∑n=1Nxn(1)νp2−zn2Izn′(ρ1)Izn(ρ1)=(5.7b)yp(1)νpα+(νp,γ)[Kνp′(ρ1)Kνp(ρ1)+Υνp(ω)Kνp(ρc)Iνp(ρc)Iνp′(ρ1)Kνp(ρ1)],p=(1),2,3,…,P,(5.7c)∑n=1Nxn(1)μk2−zn2=yk(2)μkα−(μk,γ)+aμk(0)Iμk(ρ1)μkα−(μk,γ),k=(1),2,3,…,K,(5.7d)∑n=1Nxn(1)μk2−zn2Izn′(ρ1)Izn(ρ1)=yk(2)μkα−(μk,γ)Kμk′(ρ1)Kμk(ρ1)+aμk(0)Iμk′(ρ1)μkα−(μk,γ),k=(1),2,3,…,K.

Here, the numbering of indexes started from k,p=1 for the D- and k,p=2 for the N-problem,


$$\begin{array}{c} x_{n}^{\left(1\right)}=q(z_{n},\gamma ){\bar{x}}_{n}^{\left(1\right)}. \end{array}$$

The linear algebraic systems (equation 5.7) for N-problem should be supplemented by four equations. For our convenience, let us represent them as


(5.8a)∑n=1Nxn(1)1/4−zn2=y1(1)ν1α+(1/2,γ)[1+Υ1/2(ω)K1/2(ρc)I1/2(ρc)I1/2(ρ1)K1/2(ρ1)]−x¯0(1)ν1α+(1/2,γ),p=1,∑n=1Nxn(1)1/4−zn2Izn′(ρ1)Izn(ρ1)==y1(1)ν1α+(1/2,γ)[K1/2′(ρ1)K1/2(ρ1)+Υ1/2(ω)K1/2(ρc)I1/2(ρc)I1/2′(ρ1)K1/2(ρ1)]−(5.8b)x¯0(1)ν1α+(1/2,γ)I1/2′(ρ1)I1/2(ρ1),p=1,(5.8c)∑n=1Nxn(1)1/4−zn2=y1(2)μ1α−(1/2,γ)−x¯0(1)μ1α−(1/2,γ)+π2(1+tg2γ/2)μ1α−(1/2,γ)I1/2(ρ1),k=1,∑n=1Nxn(1)1/4−zn2Izn′(ρ1)Izn(ρ1)=y1(2)μ1α−(1/2,γ)K1/2′(ρ1)K1/2(ρ1)−x¯0(1)μ1α−(1/2,γ)I1/2′(ρ1)I1/2(ρ1)+(5.8d)π2(1+tg2γ/2)μ1α−(1/2,γ)I1/2′(sc1),k=1.

Here, equation (5.8) directly follow from equation (5.6) if j=1 and involve the piston modes which do not depend on θ and uniformly spread on space. It is worth noting that $\alpha^{+}(1/2,\gamma )=2\mathrm{ctg}(\gamma /2)$, $\alpha^{-}(1/2,\gamma )=-2\mathrm{tg}(\gamma /2)$.

Next, we form from the finite system of linear algebraic equations (5.7) and (5.8), the desirable infinite one, the solution of which belongs to the required class of sequences. For this purpose, let us eliminate the unknown coefficients $y_{p}^{\left(1\right)}$, $y_{k}^{\left(2\right)}$ and ${\bar{x}}_{0}^{\left(1\right)}$ from their right-hand parts and introduce the growing sequences as


(5.9) $${\left\{\xi_{q}\right\}}_{q=1}^{N}=\left\{ \begin{array}{ll} {\left\{\nu_{p}\right\}}_{p=1}^{P}⋃{\left\{\mu_{k}\right\}}_{k=1}^{K}, & \text{ D-problem, }\\ \{1/2\}⋃{\left\{\nu_{p}\right\}}_{p=2}^{P}⋃{\left\{\mu_{k}\right\}}_{k=2}^{K}, & \text{ N-problem. } \end{array}\right.$$

Then, using the Mehler–Dirichlet integral representation of the Legendre functions [36], we transform equation (5.5) to the homogeneous Abel equation, which allows only for the trivial solution. This transforms the Legendre series (5.5) into the Dirichlet one as


(5.10) $$\begin{array}{lcr} & \lim_{J\to \infty }\;\sum_{j=1}^{J}\left[\alpha_{j}e^{\eta_{j}z}+\beta_{j}e^{-\eta_{j}z}\right]=0. & \end{array}$$

Here, $\alpha_{j}=\beta_{j}=\Lambda_{j}/2$; z=iθ if θ∈[0,γ) with $\eta_{j}=\nu_{j}$ and z=i(π−θ) if θ∈(γ,π] with $\eta_{j}=\mu_{j}$ for D- and N-problems. Further, we formulate the following theorem.

**Theorem 5.2*.*** *Equation (5.10)*, *that is formed by the series of exponents *$\left\{e^{\pm \eta_{j}z}\right\}$*, and the equation (5.5), presented in form of the Legendre functions series, allows only for the trivial solutions if *J→∞*. The proof of this theorem is presented in appendix A.*

Let us arrange our finite system of linear algebraic equations according to the sequences (5.9), and based on the statement of theorem 5.2, we pass to the limit P,K,N→∞ with N=P+K (N=P+K−1 for N-problem). As a result we arrive at ISLAE as


(5.11) $$\begin{array}{lcr} & (A_{11}+B)X^{\left(1\right)}=F^{\left(1\right)}. & \end{array}$$

Here, $X^{\left(1\right)}=\{x_{n}^{\left(1\right)}{\}}_{n=1}^{\infty }$; $A_{11}$, B are infinite matrix operators


(5.12) $$\begin{array}{lcr} & A_{11}:{\left\{a_{qn}^{\left(11\right)}=\frac{\rho_{1}W{\left[K_{\xi_{q}}I_{z_{n}}\right]}_{\rho_{1}}}{\left(\xi_{q}^{2}-z_{n}^{2}\right)K_{\xi_{q}}(\rho_{1})I_{z_{n}}(\rho_{1})}\right\}}_{q,n=1}^{\infty }, & \end{array}$$

(5.13a)B:{bqn}q,n=1∞,(5.13b)bqn=Υξq(ω)ρ1W[IξqIzn]ρ1(ξq2−zn2)Iξq(ρ1)Izn(ρ1)(Kξq(ρc)Kξq(ρ1)Iξq(ρ1)Iξq(ρc))ifξq∈{νp}p=1∞

for D-case q≥1,n≥1; for N one q>1,n≥1 and if $q=1,\text{then}\;b_{1n}$ takes the form


$$\begin{array}{lcr} & b_{1n}=-\frac{\cos^{2}(\gamma /2)\Upsilon_{1/2}(\omega )\rho_{1}W{\left[I_{1/2}I_{z_{n}}\right]}_{\rho_{1}}}{\left(1/4-z_{n}^{2}\right)I_{1/2}\left(\rho_{1}\right)I_{z_{n}}\left(\rho_{1}\right)}\left(\frac{K_{1/2}\left(\rho_{c}\right)}{K_{1/2}\left(\rho_{1}\right)}\frac{I_{1/2}\left(\rho_{1}\right)}{I_{1/2}\left(\rho_{c}\right)}\right); & \text{(5.13c)}\\ & b_{qn}=0\text{ if }\xi_{q}\in {\left\{\mu_{k}\right\}}_{k=1}^{\infty } & \text{(5.13d)} \end{array}$$

for D- and N-problems; W[⋅] is the Wronskian, $W[\alpha \beta {]}_{o}=\beta^{\prime }(o)\alpha (o)-\beta (o)\alpha^{\prime }(o)$; $F^{\left(1\right)}=\{f_{q}^{\left(1\right)}{\}}_{q=1}^{\infty }$ is the known vector defined as


(5.14a)fq(1)=π32{0,ξq∈{νk},−Pξq−12(cos⁡γ)cos⁡(πξq)Kξq(sc1),ξq∈{μk},D-problem,(5.14b)fq(1)=π32{−tg(γ/2)πK1/2(sc1),ξq=12,0,ξq∈{νp},N-problem.−Pξq−121(cos⁡γ)cos⁡(πξq)Kξq(sc1),ξq∈{μk},

Note, if P and K in growing sequences (5.9) are set to infinity, the ratio P/K=γ/(π−γ) should be valid. This truncation rule directly follows from the asymptotic formulas (A 4) (see appendix A) and is an analogy of the well-known one for the bifurcation of the parallel-plate waveguides [37]. From the physical point of view, the matrix operator $A_{11}$ in (5.11) corresponds to the wave scattering from the semi-infinite truncated cone $Q_{tr}:\{r\in (c_{1},\infty ),\theta =\gamma ,\varphi \in [0,2\pi )\}$, and the matrix operator B describes the waves scattering from the sphere-conical cavity $Q_{cav}:\left\{(r,\theta ,\phi )|c_{1}<r<c;\theta \in [0,\gamma ),\varphi \in [0,2\pi )\right\}$.

## 6. Analytical regularization

Considering the asymptotic properties of the modified Bessel and Macdonald functions, we find from (5.12) and (5.13) that


(6.1) $$a_{qn}^{\left(11\right)}=\frac{1}{\xi_{q}-z_{n}}+\left\{ \begin{array}{ll} O\left({\left\{\xi_{q}z_{n}\left(\xi_{q}-z_{n}\right)\right\}}^{-1}\right) & \text{ if }z_{n},\xi_{q}\gg \left|sc_{1}\right|\\ O\left({\left(sc_{1}/2\right)}^{\varepsilon }\right) & \text{ if }\left|sc_{1}\right|\to 0, \end{array}\right.$$

where ε=2 for D- if q,n≥1 and q>1, n≥1 for N-problem; ε=1 for N one if q=1, n≥1;


(6.2) $$b_{qn}=\left\{ \begin{array}{ll} O\left(\delta^{2\xi_{q}}/\left(\xi_{q}+z_{n}\right)\right) & \text{ if }\xi_{q}\in {\left\{\nu_{p}\right\}}_{p=1}^{\infty }\text{ and }z_{n},\xi_{q}\gg \left|sc_{1}\right|\\ 0 & \text{ if }\xi_{q}\in {\left\{\mu_{k}\right\}}_{k=1}^{\infty } \end{array}\right.\text{ D- and N-problems. }$$

Let us introduce the operator formed with the main part of the asymptotic of the matrix elements (6.1) as


(6.3a)A:{aqn=⟨ξq−zn⟩−1}q,n=1∞

and inverse one


(6.3b)A−1:{τkq=⟨{M−−1(ξq,γ)}′M−′(zk,γ)(zk−ξq)⟩−1}k,q=1∞.

Here, it will be proved that


(6.4a)A−1A=I,(6.4b)AA−1=I,

where **I **is the identity matrix; $M_{-}^{\prime }(z_{k},\gamma )=\partial_{\nu }{[M_{-}(\nu ,\gamma )]|}_{\nu =z_{k}}$, $\{[M_{-}(\xi_{q},\gamma ){]}^{-1}{\}}^{\prime }=\partial_{\nu }{[M_{-}(\nu ,\gamma ){]}^{-1}|}_{\nu =\xi_{q}}$, where $M_{-}(\nu ,\gamma )$ is determined from the factorization of the even meromorphic function M(ν,γ), which is regular in the strip Π:{|Re(ν)|<1/2} with simple zeros $\nu =\pm z_{k}$ and poles at $\nu =\pm \xi_{q}$ that are located at the real axis outside of the Π:


(6.5) $$M(\nu ,\gamma )=\pi^{-1}\cos(\pi \nu )\left\{ \begin{array}{ll} {\left\{P_{\nu -1/2}(\cos\gamma )P_{\nu -1/2}(-\cos\gamma )\right\}}^{-1}, & \text{ D-problem }\\ -{\left\{P_{\nu -1/2}^{1}(\cos\gamma )P_{\nu -1/2}^{1}(-\cos\gamma )\right\}}^{-1}, & \text{ N-problem }. \end{array}\right.$$

Here, $M(\nu ,\gamma )=M_{+}(\nu ,\gamma )M_{-}(\nu ,\gamma )$; $M_{+}(\nu ,\gamma )$, $M_{-}(\nu ,\gamma )$ are split functions regular and do not equal zero in the right (Re(ν)>−1/2) and the left (Re(ν)<1/2) half planes, respectively; $M(\nu ,\gamma )=O(\nu^{\pm 1})$ and $M_{+}(\nu ,\gamma )=M_{-}(-\nu ,\gamma )=O(\nu^{\pm 1/2})$ if |ν|→∞ in the regularity regions, where the upper sign corresponds to D- and lower to N-problems. Let us represent the split functions $M_{+}(\nu ,\gamma )$ and $M_{-}(\nu ,\gamma )$ using the infinite Weierstrass product as


(6.6) $$\begin{array}{lcr} & M_{\pm }(\nu ,\gamma )=B_{0}{\left\{\Gamma \left(\nu \pm 1/2\right)e^{\pm \chi \nu }\prod_{n=1}^{\infty }\left(1\pm \frac{\nu }{\nu_{n}}\right)e^{\mp \frac{\nu }{\nu_{n}}}\prod_{n=1}^{\infty }\left(1\pm \frac{\nu }{\mu_{n}}\right)e^{\mp \frac{\nu }{\mu_{n}}}\right\}}^{-1}. & \end{array}$$

Here, $\nu_{n}$, $\mu_{n}$ are the solutions of the transcendental (3.3a) for the D- and (3.3b) for the N-problem, respectively:


(6.7)B0=ς−1/2,(6.8)χ=γπln⁡γπ+π−γπln⁡π−γπ−ψ(α)−S(γ)−S(π−γ),(6.9)S(γ)=∑n=1∞{γπ(n−β)−1νn},S(π−γ)=∑n=1∞{π−γπ(n−β)−1μn},

where Γ(⋅) and ψ(⋅) are the Gamma function and its logarithmic derivative, respectively;


$$\begin{array}{rl} \varsigma & =P_{-1/2}(\cos\gamma )P_{-1/2}(-\cos\gamma )\text{ and }\alpha =3/4,\beta =1/4,\;\text{ D-problem, }\\ \varsigma & =-P_{-1/2}^{1}(\cos\gamma )P_{-1/2}^{1}(-\cos\gamma )\text{ and }\alpha =1/4,\beta =3/4,\text{ N-problem. } \end{array}$$

In order to prove the equalities (6.4a) and (6.4b), let us represent (6.4a) as


(6.10) $$\begin{array}{lcr} & \sum_{q=1}^{\infty }\tau_{kq}a_{qn}=\delta_{k}^{n}, & \end{array}$$

where $\delta_{k}^{n}$ is the Kronecker symbol. Let us introduce the integral as follows:


$$\begin{array}{c} J_{kn}=\frac{1}{2\pi iM_{-}^{\prime }(z_{k},\gamma )}\oint_{C_{R}}\frac{M_{-}(t,\gamma )dt}{\left(z_{k}-t)(t-z_{n}\right)}. \end{array}$$

Here, the integration path in the complex plane t is the circle $C_{R}$; the point t=0 and R are the centre and the radius of the circle, respectively; $C_{R}$ outline encompasses the simple poles of the integrand at $t=\xi_{q}$ (q=1,2,3,…) and $t=z_{k}$ if k=n. For |t|→∞, the integrand as a function of t tends to zero not slower than $t^{-3/2}$ ($t^{-5/2}$ for R-case), therefore, $J_{kn}\to 0$ if R→∞ for S- and R-cases. Then, applying the residues theorem, we arrive at the representations (6.10) and (6.4a). Next, we represent the equality (6.4b) in the form


(6.11) $$\begin{array}{lcr} & \sum_{k=1}^{\infty }a_{pk}\tau_{kn}=\delta_{p}^{n}. & \end{array}$$

Then, introducing the integral as


$$\begin{array}{c} J_{pn}=\frac{1}{2\pi i[M_{-}^{-1}(\xi_{n}){]}^{\prime }}\oint_{C_{R}}\frac{dt}{M_{-}(t)(t-\xi_{n})(\xi_{p}-t)} \end{array}$$

we prove the relation (6.11) and (6.4b) using the technique we applied in the previous case.

### (a) Left-side regularization

Using the ISLAE of the first kind (5.11) and taking into account the properties of the operators (6.4a), we formulate the original diffraction problem via the ISLAE of the second kind as follows:


(6.12) $$\begin{array}{lcr} & {X=A}^{-1}(A-A_{11})X-A^{-1}\mathrm{BX}+A^{-1}F_{1}. & \end{array}$$

The ISLAE (6.12) admits the solution in the class of sequences


(6.13) $$b(\sigma ):=\{\|X\|=\underset{n\to \infty }{\sup}|x_{n}|,\;\lim_{n\to \infty }|x_{n}n^{\sigma }|=0\},$$

where 0≤σ<3/2 for D- and 0≤σ<1/2 for N-problems, respectively. This fulfils the necessary conditions for the existence of a unique solution of the ISLAE (6.12), including the Meixner condition on the edge. The proof of these statements is based on the use of the expressions (6.1) and (6.2) as well as on the asymptotic estimate of the expression (6.3b):


(6.14) $$\tau_{kq}=O\left(\xi_{q}^{\pm 1/2}z_{k}^{\mp 1/2}/\left(z_{k}-\xi_{q}\right)\right)\text{ if }k,p\to \infty ,$$

where the upper sign corresponds to D- and lower to N-problems.

### (b) Right-side regularization

Let us introduce the new unknown vector $\overset{...}{X}$ as


(6.15) $$\begin{array}{lcr} & X=A^{-1}\overset{...}{X}. & \end{array}$$

Considering this and taking into account the equality (6.4b), let us rewrite the ISLAE (5.11) in the form of


(6.16) $$\begin{array}{lcr} & \overset{...}{X}=(A-A_{11})A^{-1}\overset{...}{X}-BA^{-1}\overset{...}{X}+F_{1}. & \end{array}$$

Equation (6.16) is the desirable ISLAE of the second kind. Therefore, the ISLAE (6.16), as well as the ISLAE (6.12), allow for obtaining the solution with the given accuracy for any geometrical and frequency parameters except the spectral ones; these solutions guarantee the fulfilment of all the necessary conditions, including the edge condition.

## 7. Family of regularizing operators

In this section, we generalize our theory and introduce the family of regularizing operators that can simplify the analysis. Let there be a given set of meromorphic functions with respect to the argument ν:


(7.1) $$\begin{array}{lcr} & ℜ:\{\overset{⌢}{M}(\nu ,\gamma )=\overset{⌢}{Q}(\nu ,\gamma )/\overset{⌢}{P}(\nu ,\gamma )\}, & \end{array}$$

where $\overset{⌢}{Q}(\nu ,\gamma )$ and $\overset{⌢}{P}(\nu ,\gamma )$ are integers, even functions of the exponential type that are not equal to zero at ν=0. We shall denote the simple zeros $\{{\overset{⌢}{z}}_{n}{\}}_{n=1}^{\infty }$ and poles $\{{\overset{⌢}{\xi }}_{q}{\}}_{q=1}^{\infty }$ of the function (7.1), where ${\overset{⌢}{z}}_{n}$ and ${\overset{⌢}{\xi }}_{q}$ are the linear functions of indices and satisfy the following asymptotic estimations:


(7.2) $$\left|z_{n}-{\hat{z}}_{n}\right|=O(1/n),\left|\xi_{q}-{\hat{\xi }}_{q}\right|=O(1/q)\text{ if }n,q\to \infty .$$

Here, $z_{n}$ and $\xi_{q}$ are simple zeros and poles of functions (6.5). Considering this, we shall form the family of couples of matrix operators $ℑ:\left\{\overset{⌢}{A},{\overset{⌢}{A}}^{-1}\right\}$ whose elements are written using the function (7.1) as those represented in (6.3a) and (6.3b), respectively. Further, using the matrix operator (6.3a), we formally introduce the set of equations


(7.3) $$\begin{array}{lcr} & \mathrm{AX}=F, & \end{array}$$

where $F:\{f_{q}{\}}_{q=1}^{\infty }$ is the known vector. Then, let us formulate the following statement.

**Theorem 7.1*.*** *An arbitrary couple of operators from* ℑ *is the regularizing one for ISLAE* (5.11).

**Proof of theorem**. Let us apply the regularization procedure (6.12) to equation (7.3) using the operators from ℑ. This leads to the ISLAE of the second kind, which we write as


(7.4) $$\begin{array}{lcr} & x_{n}+\sum_{p=1}^{\infty }c_{np}x_{p}=l_{n}. & \end{array}$$

Here,


$$\begin{array}{r} c_{np}=\sum_{k=1}^{\infty }{\hat{\tau }}_{nk}\frac{\left({\hat{\xi }}_{k}-\xi_{k}\right)+\left(z_{p}-{\hat{z}}_{p}\right)}{\left({\hat{\xi }}_{k}-{\hat{z}}_{p}\right)\left(\xi_{k}-z_{p}\right)},\\ l_{n}=\sum_{k=1}^{\infty }{\hat{\tau }}_{nk}f_{k}<\infty , \end{array}$$

${\overset{⌢}{\tau }}_{np}$ is the matrix elements of the operator ${\overset{⌢}{A}}^{-1}$, whose asymptotic behaviour is determined by the expression (6.14).

Accounting for the relations (6.14), we establish by direct verification that $c_{np}\to 0$ if n,p→∞ not slower than $n^{-\overset{⌢}{\sigma }}p^{-\overset{⌢}{\delta }}$, where $\overset{⌢}{\sigma }=3/2$, $\overset{⌢}{\delta }=3/2-\varepsilon$ for D- and $\overset{⌢}{\sigma }=1/2$, $\overset{⌢}{\delta }=2$ for N-problem with ε>0 no matter how small. Therefore, the couples of operators $\overset{⌢}{A}$, ${\overset{⌢}{A}}^{-1}$ from ℑ are regularizing for equation (7.3). It follows directly from this that couples of operators from ℑ are also regularizers for ISLAE (5.11).∎

*Examples of the new regularizing operators*. Let us introduce the function $\overset{⌢}{M}(\nu )$ with the simple zeros ${\overset{⌢}{z}}_{n}=z_{n}$ and the poles ${\overset{⌢}{\xi }}_{q}$ that coincide with the main parts of asymptotic of the indexes $\xi_{q}$ if q→∞ (γ≠π/2). Taking into account the asymptotic properties of the Legendre and associated Legendre functions, we find that


$$\begin{array}{c} {\overset{⌢}{\xi }}_{q}\in {\left\{{\overset{⌢}{\nu }}_{p}=\pi (p-1/4)/\gamma )\right\}}_{p=1}^{\infty }⋃{\left\{{\overset{⌢}{\mu }}_{k}=\pi \;(k-1/4)/(\pi -\gamma )\right\}}_{k=1}^{\infty },\;q=1,2,3,\ldots ,\text{D-problem},\\ {\overset{⌢}{\xi }}_{q>1}\in {\left\{{\overset{⌢}{\nu }}_{p}=\pi (p+1/4)/\gamma )\right\}}_{p=1}^{\infty }\;⋃{\left\{{\overset{⌢}{\mu }}_{k}=\pi \;(k+1/4)/(\pi -\gamma )\right\}}_{k=1}^{\infty },\;q=2,3,4,\ldots ,\text{N-problem}, \end{array}$$

and ${\overset{⌢}{\xi }}_{1}=\xi_{1}=1/2$ for N-problem. Then, $\overset{⌢}{M}(\nu )$ is expressed through Gamma functions as


$$\begin{array}{lrlr} & & \overset{⏜}{M}(\nu ,\gamma )=\frac{\Gamma \left(\frac{3}{4}+\frac{\gamma }{\pi }\nu \right)\Gamma \left(\frac{3}{4}-\frac{\gamma }{\pi }\nu \right)\Gamma \left(\frac{3}{4}+\frac{\pi -\gamma }{\pi }\nu \right)\Gamma \left(\frac{3}{4}-\frac{\pi -\gamma }{\pi }\nu \right)}{\Gamma \left(\frac{1}{2}+\nu \right)\Gamma \left(\frac{1}{2}-\nu \right)},\text{ D-problem, } & \text{(7.5a)}\\ & & \overset{⏜}{M}(\nu ,\gamma )=\frac{\Gamma \left(\frac{5}{4}+\frac{\gamma }{\pi }\nu \right)\Gamma \left(\frac{5}{4}-\frac{\gamma }{\pi }\nu \right)\Gamma \left(\frac{5}{4}+\frac{\pi -\gamma }{\pi }\nu \right)\Gamma \left(\frac{5}{4}-\frac{\pi -\gamma }{\pi }\nu \right)}{{\left(\frac{1}{2}+\nu \right)}^{2}{\left(\frac{1}{2}-\nu \right)}^{2}\Gamma \left(\frac{1}{2}+\nu \right)\Gamma \left(\frac{1}{2}-\nu \right)},\text{ N-problem. } & \text{(7.5b)} \end{array}$$

Functions (7.5) are even and regular in the strip Π; $\overset{⌢}{M}(\nu ,\gamma )=O(\nu^{\pm 1})$ if |ν|→∞, where the upper sign corresponds to D- and the lower to N-problem. These functions elementarily factorize and are written as


(7.6) $$\begin{array}{lcr} & \overset{⌢}{M}(\nu ,\gamma )={\overset{⌢}{M}}_{+}(\nu ,\gamma ){\overset{⌢}{M}}_{-}(\nu ,\gamma ), & \end{array}$$

where


(7.7a)M⌢±(ν,γ)=e∓νχ^Γ(34±γπν)Γ(34±π−γπν)Γ(12±ν), D-problem, (7.7b) M⌢±(ν,γ)=e∓νχ^Γ(54±γπν)Γ(54±π−γπν)(12±ν)2Γ(12±ν), N-problem. 

Here, ${\overset{⌢}{M}}_{+}(\nu ,\gamma ),{\overset{⌢}{M}}_{-}(\nu ,\gamma )$ are regular functions in the semi-planes Re(ν)>−1/2, Re(ν)<1/2, respectively, and ${\overset{⌢}{M}}_{+}(\nu ,\gamma )={\overset{⌢}{M}}_{-}(-\nu ,\gamma )=O(\nu^{\pm 1/2})$, if |ν|→∞ in the regularity regions, where the upper sign corresponds to D- and the lower to N-problems;


(7.8) $$\hat{\chi }=\frac{\gamma }{\pi }\ln\frac{\gamma }{\pi }+\frac{\pi -\gamma }{\pi }\ln\frac{\pi -\gamma }{\pi }.$$

It is worth noting that using the kernel functions (7.5) essentially simplifies the construction of the regularizing operators $\overset{⌢}{A}$, ${\overset{⌢}{A}}^{-1}$. They allow for reducing the problem to the ISLAE of the second kind, whose solutions exist in the necessary class of sequences. However, their effectiveness is smaller than the initial regularizing operators because these new operators explicitly inverted only the main part of the asymptotic of the operator (6.3a) for D- and N-problems.

## 8. Excitation of the sphere-conical resonator through the small circular hole

The main idea of our analysis in this section is to obtain an expression for determining the perturbation of the eigen-frequencies of a sphere-conical resonator caused by a small-size hole, which rigorously takes into account the singularity of the matrix operator (5.11) for the initial wave diffraction problem. The various approximate techniques to determine the perturbation of the spectral properties of the resonators by small-size holes were developed in [23,38–40].

Let us rewrite our basic equation (6.12) by way of


(8.1) $$\begin{array}{lcr} & x_{k}^{\left(1\right)}=\sum_{q=1}^{\infty }\tau_{kq}\sum_{n=1}^{\infty }(a_{qn}-a_{qn}^{\left(11\right)})x_{n}^{\left(1\right)}-\sum_{q=1}^{\infty }\tau_{kq}\sum_{n=1}^{\infty }b_{qn}x_{n}^{\left(1\right)}+\phi_{k},k=1,2,3,\ldots & \end{array}$$

where


(8.2) $$\begin{array}{lcr} & \phi_{k}=\sum_{q=1}^{\infty }\tau_{kq}f_{q}^{\left(1\right)}. & \end{array}$$

Let us simplify equation (8.1). For this purpose, we consider the small dimensions of the hole ($|\rho_{1}/2|<<1$). Thus, we apply the appropriate asymptotic expressions for modified Bessel and Macdonald functions to estimate the known coefficients in equation (8.1). Then, taking into account the asymptotic estimation (6.1), we neglect the dynamic terms in the first double series. This leads to the approximate equation as


(8.3) $$\begin{array}{lcr} & x_{k}^{\left(1\right)}+2\sum_{q=1}^{\infty }\tau_{kq}\kappa_{\xi_{q}}(\rho_{c})y_{q}{\left(\frac{\rho_{1}}{2}\right)}^{2\xi_{q}}=\phi_{k},\;\;k=1,2,3,\ldots , & \end{array}$$

where


(8.4) $$\begin{array}{lcr} & y_{q}=\sum_{n=1}^{\infty }\frac{x_{n}^{\left(1\right)}}{\xi_{q}+z_{n}}, & \end{array}$$

(8.5a)κξq(ρc)={Θ1(ξq)Kξq(ρc)Iξq(ρc) if ξq∈{νp}p=1∞,0 if ξq∈{μk}k=1∞, D-problem, (8.5b)κξq(ρc)={Kξq(ρc)−2ρcKξq′(ρc)Iξq(ρc)−2ρcIξq′(ρc)(Θ1(ξq>1)Θ1(ξq=1)) if ξq∈{1/2}⋃{νp}p=2∞,0 if ξq∈{μk}k=2∞ N-problem. 

Here, $\rho_{c}=-i\omega c/\upsilon$, $\Theta_{1}(\xi_{q>1})=[\Gamma (\xi_{q})\Gamma (\xi_{q}+1){]}^{-1}$, $\Theta_{1}(\xi_{q=1})=2\pi^{-1}\cos^{2}(\gamma /2)$. Note that the simple poles of the functions (8.5) correspond to the resonance oscillation of the closed sphere-conical resonator.

Let us consider the resonant excitation of cavity with the small size hole. Taking into account the time factor $e^{-iwt}$, the real and imaginary parts of the resonance frequency of an open sphere-conical cavity $w_{res}=Re(w_{res})+iIm(w_{res})$ are determined as $Re(w_{res})>0$, $Im(w_{res})\leq 0$. Under these conditions, the resonance frequency has a physical sense, i.e. the wave moves from the scatterer to infinity, the corresponding vibration grows in volume and decreases with time.

Let us introduce ${\bar{\rho }}_{res}=-i\omega_{\xi_{qj}}c/\upsilon$ (q,j=1,2,3,…), where $\omega_{\xi_{qj}}$ is the real jth resonant frequency of the axially symmetric $\xi_{q}$-mode of the closed resonator (Im(ωξqj)=0); ${\bar{\rho }}_{res}$ is determined from the solution of the transcendental equations as


(8.6a)Iξq(ρ¯res)=0, D-problem, (8.6b)Iξq(ρ¯res)−2ρ¯resIξq′(ρ¯res)=0, N-problem. 

Let $\rho_{c}={\bar{\rho }}_{res}+\Delta \rho_{c}$, where $\Delta \rho_{c}\equiv -i\Delta \omega_{\xi_{qj}}c/\upsilon$; $\Delta \omega_{\xi_{qj}}$ is the perturbation of the resonant frequency $\omega_{\xi_{qj}}$ for the closed resonator, where the lower index j shows the number of the root of equation (8.6), Δωξqj=ReΔωξqj+iImΔωξqj and $|\Delta \rho_{c}|<<1$. Under these conditions and if $|\rho_{1}/2|<<1$, the further simplification of the ISLAE (8.3) leads to


(8.7) $$\begin{array}{lcr} & x_{k}^{\left(1\right)}+2\tau_{kq}\kappa_{\xi_{q}}(\rho_{c})y_{q}{\left(\frac{\rho_{1}}{2}\right)}^{2\xi_{q}}=\phi_{k}. & \end{array}$$

Here, k=1,2,3,…, index $\xi_{q}$ corresponds to a resonantly excited mode of the closed resonator. Therefore, the small parameter $|\rho_{1}/2|$ in equation (8.3) is kept only at the resonant term. From equation (8.7), we derive the correlation as


(8.8) $$\begin{array}{lcr} & x_{n}^{\left(1\right)}\tau_{kq}-x_{k}^{\left(1\right)}\tau_{nq}=\phi_{n}\tau_{kq}-\phi_{k}\tau_{nq}, & \end{array}$$

where k≠n. Taking this into account, we represent expression (8.4) as


(8.9) $$\begin{array}{lcr} & y_{q}=\frac{x_{k}^{\left(1\right)}}{\tau_{kq}}h(\xi_{q})-\frac{\phi_{k}}{\tau_{kq}}h(\xi_{q})+d(\xi_{q}). & \end{array}$$

Here, k,q=1,2,3,…,


(8.10a)d(ξq)=∑n=1∞ϕnξq+zn,(8.10b)h(ξq)=∑n=1∞τnqξq+zn.

To obtain the summation formula for series (8.10b), let us introduce the integral as


(8.11) $$\begin{array}{lcr} & J_{q}=\frac{1}{2\pi i}\int_{C_{R}}\frac{1}{\left(\xi_{q}+t)(t-\xi_{q})M_{-}(t\right)}dt, & \end{array}$$

where the circle $C_{R}$ with radius R envelopes the simple poles of the integrand at $t=z_{n}$(n=1,2,3,…) and $t=-\xi_{q}$. The integrand (8.11) decays as $t^{-5/2}$ for D- and $t^{-3/2}$ for N-problems, if R→∞. Next, using the residue theorem and expression (6.3b), it is found that


(8.12) $$\begin{array}{lcr} & h(\xi_{q})=\frac{1}{2\xi_{q}[M_{-}^{-1}(\xi_{q}){]}^{\prime }M_{+}(\xi_{q})}. & \end{array}$$

Let us substitute the expressions (8.9) into (8.7) and find that


(8.13) $$\begin{array}{lcr} & x_{k}^{\left(1\right)}=\frac{\phi_{k}+2\kappa_{\xi_{q}}(\rho_{c})[\phi_{k}h(\xi_{q})-\tau_{kq}d(\xi_{q})]{\left(\frac{\rho_{1}}{2}\right)}^{2\xi_{q}}}{1+2h(\xi_{q})\kappa_{\xi_{q}}(\rho_{c}){\left(\frac{\rho_{1}}{2}\right)}^{2\xi_{q}}}. & \end{array}$$

Therefore, the expression (8.13) gives the approximate solution of the diffraction problem near the resonant excitation of $\xi_{q}$-mode if the truncation radius of the cavity is small. Equating the denominator (8.13) to zero and using the expression (8.12), we arrive at the characteristic equation to determine the resonance frequency perturbations of the sphere-conical resonator by the small circular hole as


(8.14) $$\begin{array}{lcr} & \xi_{q}[M_{-}^{-1}(\xi_{q}){]}^{{,}^{\prime }}M_{+}(\xi_{q})+\kappa_{\xi_{q}}(\rho_{c})(\rho_{1}/2{)}^{2\xi_{q}}=0. & \end{array}$$

Further, using equation (8.14) and the expressions (8.5), we find that


(8.15) $$\Delta \omega_{\xi_{qj}}=\frac{e^{-i\pi /2}v\Theta_{1}\left(\xi_{q}\right){\left(\frac{\rho_{1}}{2}\right)}^{2\xi_{q}}}{c\xi_{q}{\left[M_{-}^{-1}\left(\xi_{q}\right)\right]}^{\prime }M_{+}\left(\xi_{q}\right)}\left\{ \begin{array}{ll} \frac{K_{\xi_{q}}\left({\bar{\rho }}_{res}\right)}{\partial_{\rho }I_{\xi_{q}}\left({\bar{\rho }}_{res}\right)}, & \text{ D-problem, }\\ \frac{K_{\xi_{q}}\left({\bar{\rho }}_{res}\right)-2{\bar{\rho }}_{res}K_{\xi_{q}}^{\prime }\left({\bar{\rho }}_{res}\right)}{\partial_{\rho }\left[I_{\xi_{q}}\left({\bar{\rho }}_{res}\right)-2zI_{\xi_{q}}^{\prime }\left({\bar{\rho }}_{res}\right)\right]}, & \text{ N-problem. } \end{array}\right.$$

Here, $|\Delta \omega_{\xi_{qj}}|<<\omega_{\xi_{qj}}$. Therefore, as follows from our definition, Im(Δωξqj)<0 and Re(Δωξqj) can take any sign. From the correlation (8.15), it follows that $\Delta \omega_{\xi_{qj}}$ is a complex value and depends on the truncated dimensionless radius $\rho_{1}/2$, opening angle γ and resonant parameter of the closed sphere-conical resonator ${\bar{\rho }}_{res}$.

Note that the expression (8.15) accurately determines the resonance frequency perturbation because the field singularity at the edge of the circular hole and resonator geometry are taken into account.

## 9. Numerical calculation

All the scattered field characteristics are studied using the reduced ISLAE (6.12). We analyse near and far-fields for different geometrical and frequency parameters. For far-field analysis (r→∞), we apply the asymptotic expression as


(9.1) $$\begin{array}{lcr} & U(r,\theta )∼S(\theta )\frac{e^{ikr}}{kr}, & \end{array}$$

where


(9.2) $$\begin{array}{lcr} & S(\theta )=\sqrt{\frac{\pi }{2}}\sum_{k=1}^{\infty }\frac{y_{k}^{\left(2\right)}P_{\mu_{k}-1/2}(-\cos\;\theta )}{K_{\mu_{k}}(sc_{1})} & \end{array}$$

and the far-field pattern is defined by D(θ)=|S(θ)|.

Further, we consider the soft and rigid scatterers separately and compare their scattered and resonance properties.

### (a) Soft scatterer

Let us consider the reduced ISLAE (6.12) and analyse the corresponding finite linear algebraic equations for the D-problem. We validate the numerical analysis by using the dependencies of the relative calculation error e(N) of the near fields as


$$\begin{array}{c} e(N)=\frac{|U^{\left(N+1\right)}-U^{\left(N\right)}|}{|U^{\left(N\right)}|}100\%, \end{array}$$

where $U^{\left(N\right)}=U^{\left(N\right)}(r,\theta )$ is the field potential calculated using N×N set of linear algebraic equations, and check the mode matching.

**Figure 2.**
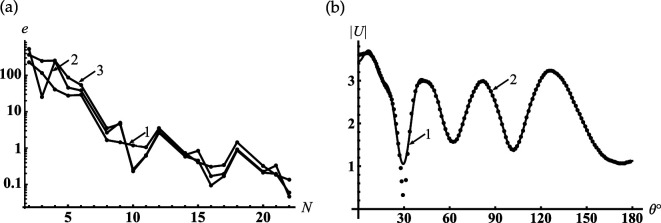
Verification of the numerical analysis for the soft cone with $\gamma =30^{\circ }$: (a) dependencies of the relative error on the truncation order for near field calculation if $kc_{1}=5$, kc=6, $kr=kc_{1}-1$; (1) θ=γ, (2) θ=(π−γ)/2, (3) θ=π; (b) testing of the mode-matching if $kc_{1}=5$, kc=6; (1) $kr=kc_{1}+0$, (2) $kr=kc_{1}-0$.

In figure 2a, we represent the typical dependencies of the relative error of the near field calculation on the truncation parameter N for fixed $kr=kc_{1}-1$ and for the three points of θ=γ,(π−γ)/2,π. We can see from this figure that the accuracy of calculations of the order of 0.1% is achieved at N=22. An accuracy of less than 0.1% can be obtained if N>34. Taking this into account, we chose the truncation parameter $N=[|sc_{1}|]+\left[q\right]$ and 4≤q≤10, where [⋅] denotes the entire part. To verify the mode-matching, we calculate |U(r,θ)| on the virtual spherical surfaces with radii $kr=kc_{1}\pm 0$, and find the excellent adjustment of the field behaviour on the above-mentioned virtual surfaces (see figure 2b). It should be noted that the curve 1 in this figure tends to zero if the observation angle θ approaches the surface of the cone $\gamma =30^{\circ }$, since it satisfies the boundary condition in the area $c_{1}+0$. This is the reason for the discrepancy between curves 1 and 2 in the vicinity of $\gamma =30^{\circ }$.

**Figure 3.**
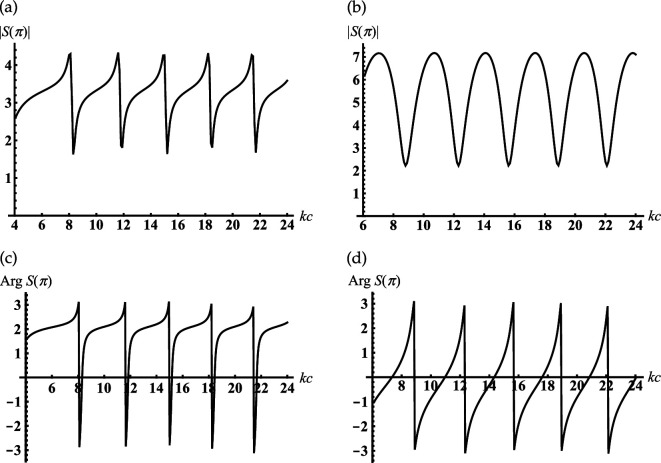
Dependencies of the backscattering of module and argument on the termination coordinate kc for soft cone with $\gamma =30^{\circ }$: (a,c) $kc_{1}=4$; (b,d) $kc_{1}=6$.


Consider an open-ended sphere-conical cavity formed by the truncated semi-infinite cone with opening angle $\gamma <90^{\circ }$ and an internal termination in the form of the spherical-cap $kc>kc_{1}$ (see figure 1a). This geometry can be used a model of open resonators, concaved spherical reflectors and probes/antennas for different measurements. In order to study its scattering properties, figure 3 represents the dependencies of the module |S(π)|=D(π) and phase ArgS(π) of the complex amplitude of the backscattering field (9.2) as the function of the dimensionless frequency parameter kc. These characteristics are calculated for different dimensionless truncation radii of the cavity $kc_{1}$. From figure 3a, it is observed that the periodical sharp jumps |S(π)| as the function of kc. The adjacent upper and lower peaks of these jumps are located close to the dimensionless resonant radii for vibrations $U_{\nu ,0,j}$ of the corresponding closed sphere-conical resonator $Q_{r}:\left\{r\in [0,c];\theta \in [0,\gamma ]\right\}$,

(9.3) $$\begin{array}{lcr} & U_{\nu ,0,j}=(k_{j}r{)}^{-1/2}J_{\nu }(k_{j}r)P_{\nu -1/2}(\cos\;\theta ), & \end{array}$$

where $\nu =\nu_{1}$; the resonant radii are determined as the positive roots of the transcendental equation $J_{\nu_{1}}(k_{j}c)=0$ (see also (8.6a); $J_{\nu }(x)$ is the Bessel function).

Some examples of dimensionless resonant radii $k_{j}c$ for closed resonator $Q_{r}$ and parameters kc for adjacent upper and lower peaks/jumps in figure 3a are represented in table 1. Comparing the curves in figure 3a,b, we see how increasing the cavity’s truncation radius $kc_{1}$ leads to changes |S(π)|. In contrast to figure 3a, the curve in figure 3b, which corresponds to the larger $kc_{1}$, shows a smooth oscillation of |S(π)|. In order to explain this, let us consider dependencies ArgS(π) on kc for different $kc_{1}$ in figure 3c,d, respectively. We observe their sharp jumps if kc approaches dimensionless resonant radii $k_{j}c$ of the corresponding closed sphere-conical resonator; the location of these jumps weakly depends on $kc_{1}$. However, the behaviour ArgS(π) between the adjacent jump maxima essentially depends on the truncation radius $kc_{1}$. We observe the wide interval of kc, where the phase is practically stable in figure 3c, and its monotonous growth throughout this interval in figure 3d. Therefore, we see from these that oscillations |S(π)| are caused by the passes kc through the cavity’s resonant radii; the influence of $kc_{1}$ on their shapes can be explained by the changes in the behaviour of ArgS(π) between the adjacent jump maxima. It is worth noting that the deep oscillations of the backscattering |S(π)| in figure 3 can be used to regulate the efficiency of radiation from the resonator.

**Table 1.** Dimensionless resonant radii of the closed soft sphere-conical cavity $Q_{r}$ for its $U_{\nu_{1},0,j}$ vibrations for the soft cone with $\gamma =30^{\circ }$ and $\nu_{1}=4.58369$; the local maxima and minima of the curve in figure 3a.

number of roots and extrema	j=1	j=2	j=3	j=4	j=5
roots of equation $J_{\nu_{1}}(k_{j}c)=0$	8.28148	11.8114	15.1507	18.4153	21.6417
kc for the local maxima	8.065	11.615	14.961	18.228	21.457
kc for the local minima	8.316	11.843	15.181	18.445	21.672

**Figure 4.**
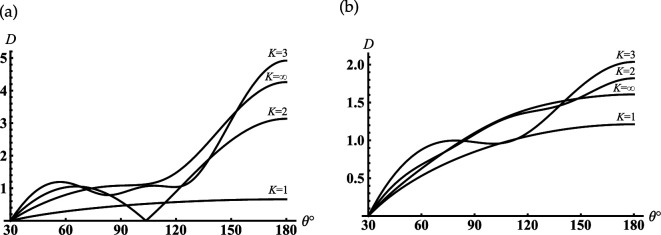
Far-field patterns for the soft cone with $\gamma =30^{\circ }$, $kc_{1}=4$: (a) kc=8.065; (b) kc=8.316 are the first local maximum and minimum of the curve in figure 3a; (1) K=1, (2) K=2, (3) K=3 (K is the number of modes that are taken into account in (9.2)); K=∞ marks the final curve.

Further, we analyse the far-field modes to understand how the resonance one radiates from the cavity’s open-end. The curves in figure 4a show the contribution of the different modes for far-field formation if the first resonance mode is excited in the sphere-conical cavity (see the first peak in figure 3a). As follows from figure 4a, the sum of the first three modes (9.2) gives the main contribution to the far field for all observation angles γ<θ<π. The mode analysis of the far field for the first minimum of the back radiation in figure 3a is shown in figure 4b. We see from this figure the same dominant modes as in the previous case. The difference between these two cases is that in the first case (see figure 4a), we observe the effective focusing of the main lobe in the back direction.

**Figure 5.**
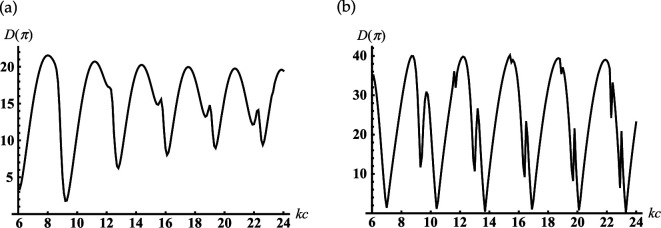
Dependencies of the backscattering on the termination coordinate kc for the soft cone with $kc_{1}=6$: (a) $\gamma =60^{\circ }$ and (b) $\gamma =90.01^{\circ }$.

In figure 5, we represent the typical oscillatory dependencies of the back radiation on the position of the internal termination for resonators with different opening angles. Comparing the curves in figures 3 and 5, we see that increasing the truncation radius $kc_{1}$ and the opening angle γ leads to the significant growth of the maxima of the back radiation.

**Figure 6.**
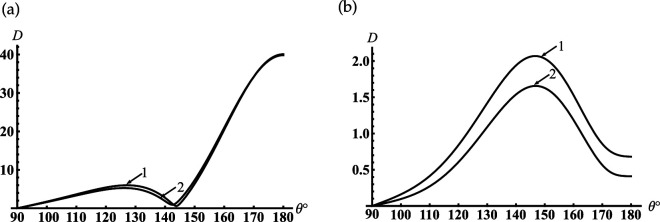
Far-field patterns scattered from the soft cone with $kc_{1}=6$, $\gamma =90.01{,}^{\circ }$ for two maxima of the curve in figure 5b: (a) kc=8.747(12.217) and minima (b) kc=10.422(13.699); curve 1 is calculated for kc=8.747 and kc=10.422 and curve 2 is calculated for kc=12.217 and kc=13.699.

Suppose the cone degenerates into the plane with the subsurface half-sphere cavity $\gamma \approx 90^{\circ }$ (see figure 1b). It is worth noting we do not apply $\gamma =90^{\circ }$ because this case requires separate consideration [41]. From figure 5b, it is observed the deep oscillations of the backscattering and the discrete dimensionless radii/frequencies kc for which D(π)→0 and the backscattering are practically entirely suppressed. This property can be applied for modelling of artificial black surfaces or selective scattering ones. Considering that the scatterer with $\gamma \approx 90^{\circ }$ can also be used to model a subsurface defect and the discrete positions of the local maxima in figure 5b can help determine the frequency of the sounding wave for its diagnostics. In figure 6, we depict the far field scattered from this scatterer. Figure 6a curves represent the field distribution for two cases that correspond to the two local maxima of the curve represented in figure 5b for kc=8.747 and kc=12.217. Curves in figure 6b represent the field distribution if kc=10.422 and kc=13.699 corresponding to the two local minima of the curve in figure 5b. We see from figure 6a that if a plane surface with a half-spherical cavity is normally illuminated by a plane wave at resonant frequencies, the scattered maxima are formed in the backscattering direction. In the opposite case (see figure 6b), the maxima of radiation are directed towards the sector $140^{\circ }<\theta <160^{\circ }$ and are formed by the radiated edge-waves.

**Figure 7.**
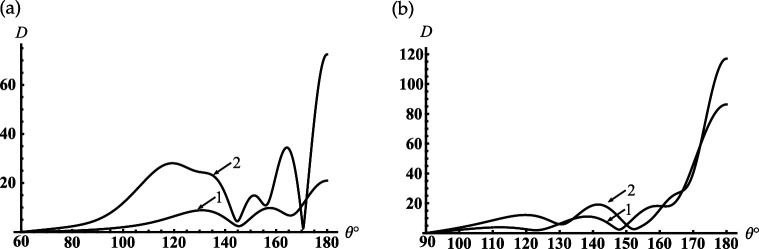
The far-field scattered from the soft sphere-conical reflector with (a) $\gamma =60{,}^{\circ }$, (b) $\gamma =90.01{,}^{\circ }$: (1) $kc_{1}=12$, kc=12.1; (2) $kc_{1}=18$, kc=18.1.

Let us consider the case if the internal termination of the cavity moves to its open end, which can be considered the model of the sphere-conical reflector (see figure 1a,b). Curves in figure 7 show the far field scattered from this reflector with the different radii of the aperture and the opening angles of the cone. Comparing the curves in figure 7a,b, we can conclude that the reflectors mentioned above focus the scattering field along the axis $\theta =180^{\circ }$. It should be noted that there is a significant increase in backscattering of a hemi-spherical reflector on a flat surface (see figure 7b).

**Figure 8.**
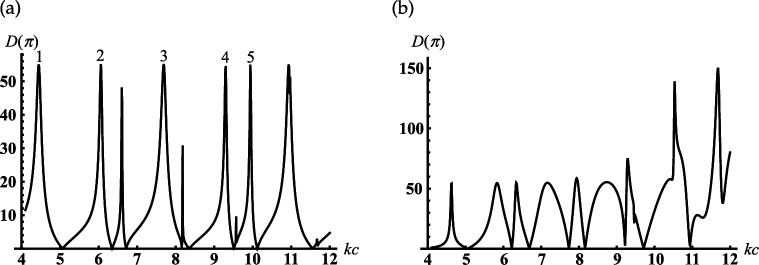
Dependencies of the backscattering on the termination coordinate kc for the soft cone with $\gamma =150^{\circ }$: (a) $kc_{1}=4$; (b) $kc_{1}=kc-1$.

If γ>π/2, our structure becomes an open spherical resonator conjoined with the semi-infinite conical horn (see figure 1c); the truncated end of the cone penetrates into the spherical volume. In figure 8a, we observe the dependence of the back radiation from this resonator into the conical horn on the dimensionless radius kc. The peaks in this figure are located close to the resonant radii $k_{j}c$ of two closed resonators: spherical and sphere-conical. Identification of the types of resonant vibrations corresponding to the selected/numbered maxima in figure 8a are presented in table 2. This table also compares the resonant radii of open and closed resonators. We see from this table that two types of spherical modes with index ν=p=1/2 (see peak numbers. 2, 4) and ν=p=3/2 (see peak numbers 1, 3) are excited in the open spherical resonator. Due to the end of the conical horn penetrating the spherical volume through its open end ($c>c_{1}$), the sphere-conical mode with index $\nu_{1}$ is also excited (see peak number 5 in figure 8a). Therefore, despite an open end, our sphere-conical structure supports the resonant vibration of two perfectly closed resonators. This case is an interesting phenomenon that shows the possibility of tuning and switching the resonant excitation modes depending on the depth of cone end penetrating into the spherical resonator. In figure 8b we represent a similar dependence if the depth of the horn end penetration into the sphere is fixed, $k(c-c_{1})=1$.

**Table 2.** Dimensionless resonant radii of $U_{\nu ,0,j}$ vibrations for closed soft spherical resonator with ν=p:(=1/2;3/2) and sphere-conical one with $\nu =\nu_{1}=0.8462$ and $\gamma =150^{\circ }$ ; $k_{j}c$ is the position of the peaks in figure 8a.

peak number in figure 8a	1	2	3	4	5
type of vibration $U_{\nu ,0,j}$	$U_{3/2,0,1}$	$U_{1/2,0,2}$	$U_{3/2,0,2}$	$U_{1/2,0,3}$	$U_{\nu_{1},0,3}$
roots of equation $J_{\nu }(k_{j}c)=0$	4.4934	6.28319	7.72525	9.4248	9.94526
$k_{j}c$	4.446	6.059	7.690	9.296	9.942

### (b) Rigid scatterer

**Figure 9.**
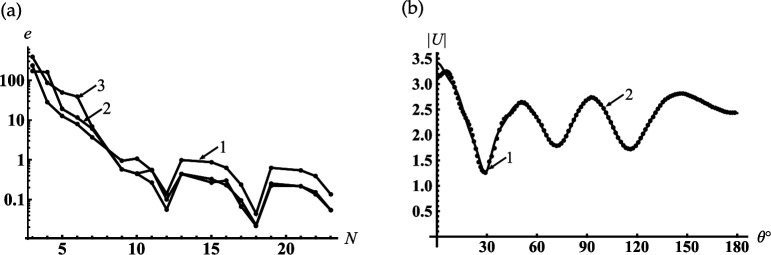
Verification of the numerical analysis for rigid cone with $\gamma =30^{\circ }$: (a) dependencies of the relative error on the truncation order for near field calculation if $kc_{1}=5$, kc=6, $kr=kc_{1}-1$; (1) θ=γ, (2) θ=(π−γ)/2, (3) θ=π; (b) testing of the mode-matching if $kc_{1}=5$, kc=6; (1) $kr=kc_{1}+0$, (2) $kr=kc_{1}-0$.

In figure 9a, we represent the typical dependencies of the relative error e(N) of the near field calculated for the rigid cone on the truncation parameter N. This figure shows that these dependencies are similar to those we saw for the soft one. An accuracy of less than 0.1% can be obtained if N>34. To verify the mode-matching, we calculate |U(r,θ)| on the virtual spherical surfaces, the radii of which are equal to $kr=kc_{1}\pm 0$, and find the excellent adjustment of the field on the above-mentioned virtual surfaces (see figure 9b).

**Figure 10.**
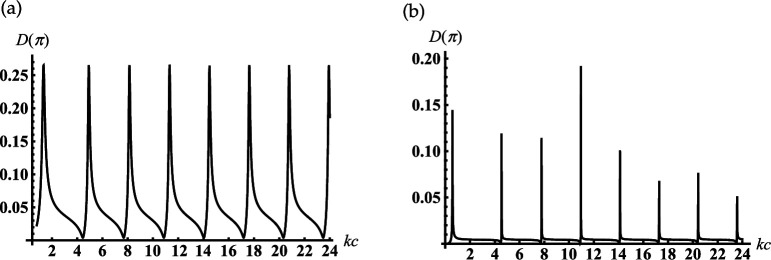
Dependencies of the backscattering on the termination coordinate kc for the rigid cone with $\gamma =30^{\circ }$: (a) $kc_{1}=0.7$, (b) $kc_{1}=0.1$ .

Let us consider an open-ended sphere-conical cavity formed by the truncated semi-infinite cone with the opening angle $\gamma <90^{\circ }$ and an internal termination in the form of the spherical-cap (see figure 1a). In figure 10, we represent the dependencies of the backscattering fields on the dimensionless frequency parameter kc, i.e. dimensionless radial coordinate of an internal termination kc, for the different truncation radii of the cavity $kc_{1}$.

We analyse the back radiation properties of the cavity with the small truncation radii $kc_{1}<1$. We see the periodic peaks of the back radiation from figure 10a,b. The position of these peaks except the first one practically does not depend on the truncation radii $kc_{1}$ for the small one. These peaks started from the second ones are located very close to the dimensionless resonant radii $k_{j}c$ of the piston mode $U_{\nu_{1}0j}$ of the corresponding rigid closed sphere-conical resonator with $\nu_{1}=1/2$ and j=0,1,2,…. Here, $k_{j}c$ is defined as the non-negative roots of the transcendental equation: $J_{1/2}(x)-2xJ_{1/2}^{\prime }(x)=0$ (see also equation (8.6b)). Examples of its roots and the local maxima of the curves in figure 10a,b are presented in table 3.

**Table 3.** Dimensionless resonant radii of the closed rigid sphere-conical cavity for its $U_{\nu_{1},0,j}$ vibrations for cone with $\gamma =30^{\circ }$ and $\nu_{1}=0.5$ ; the local maxima of the curves in figure 10a,b.

number of roots and maxima	j=0	j=1	j=2	j=3	j=4	j=5
roots of equation $J_{\nu_{1}}(x)-2xJ_{\nu_{1}}^{\prime }(x)=0$	0	4.4934	7.7252	10.904	14.066	17.630
local maxima in figure 10a	1.336	4.919	8.139	11.315	14.476	17.630
local maxima in figure 10b	0.584	4.552	7.782	10.960	14.122	17.276

**Figure 11.**
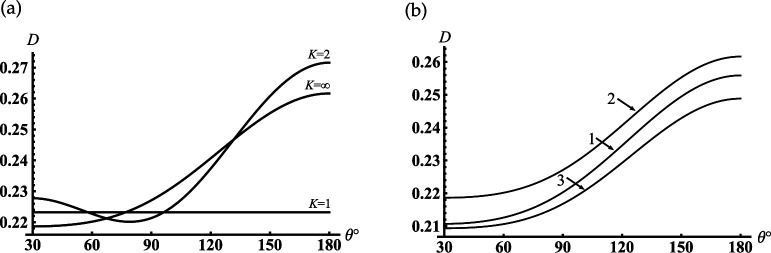
Far-field patterns for the rigid cone with $\gamma =30^{\circ }$, $kc_{1}=0.7$: (a) kc=8.14, (1) K=1, (2) K=2 (K is the number of modes that are taken into account in (9.2)); K=∞ marks the final curve; (b) (1)- kc=1.34, (2) kc=8.14, (3) kc=14.5.

As follows from figure 10a if $kc_{1}=0.7$ then the minimal resonant radius of the open cavity kc<1.5. Comparing the curves in figure 10a,b, we see that the first peak shifts to zero if the truncation radius of the cavity decreases. The curves in figure 11a show the contribution of the radiated modes for far-field formation under the condition that the third resonance peak in figure 10a is excited. Figure 11a shows that two radiated modes practically form the far field distribution. The lowest piston mode (see curve 1) gives the dominant contribution in this figure. The maximum of the relative difference between the curve corresponds to the contribution of the final distribution marked K=∞, which is reached if $\theta \to 180^{\circ }$ and the piston mode marked K=1 is less than 15%. It is worth noting that the piston mode, which is excited in the open-ended cavity $D_{1}$ is also the eigen-lowest mode for the spherical $D_{2}$ and conical $D_{3}$ regions; the two last regions $D_{2}$ and $D_{3}$ complement the region $D_{1}$ to the whole space (see definitions (2.2)). Thus, the first peaks in figure 10a,b can be explained as piston mode resonances excited due to the coupling of the open-ended cavity to the open conical area and can be considered as the low-frequency resonance of the whole dynamic system. Three curves in figure 11b show far-field radiation patterns that correspond to three different resonant peaks of the piston mode in figure 10a. This figure shows that the excitation of the different resonance regimes for the lowest cavity mode forms the far diffracted field practically with the same shapes. This fact can be considered as confirmation of the same mechanism of transformation of resonant piston modes by the cavity edge.

To summarize the above, the open rigid sphere-conical resonator $D_{1}$ effectively radiates the acoustic piston wave through the small-size hole ($kc_{1}<1$) under the resonant condition of its excitation. The small size of an open end allows for the lowest-frequency piston wave resonance caused by the coupling of an open cavity to the open conical area.

**Figure 12.**
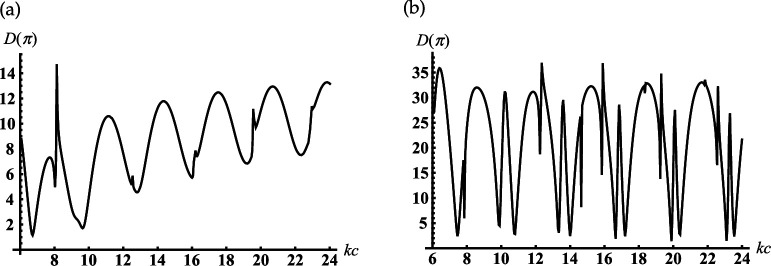
Dependencies of the backscattering on the termination coordinate kc for the rigid cone with $kc_{1}=6$: (a) $\gamma =60^{\circ }$;(b) $\gamma =90.01^{\circ }$.

In figure 12, we represent the typical oscillatory dependencies of the back radiation on the position of an internal termination for cavities with different opening angles. Comparing the curves in figures 10 and 12, we see that increasing the truncation radius $kc_{1}$ and the opening angle γ leads to the significant growth of the maxima of the back radiation. This effect is similar to that we observe above for the soft scatterer.

If the cone degenerates into the plane $\gamma \approx 90^{\circ }$ with the subsurface half-sphere cavity (see figure 1b), we observe deep oscillations of the curve in figure 12b. Therefore, we can find the discrete dimensionless cavity radii/frequencies kc for which the backscattering is effectively suppressed in this figure. This fact, similar to that we analyse above for the soft scatterer, can be used for modelling artificial surfaces and the local maxima in this figure can help determine the frequency of the sounding wave for detection of the subsurface defects.

**Figure 13.**
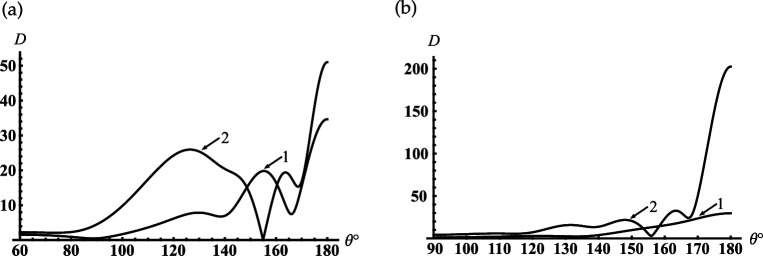
The far field scattered from the rigid sphere-conical reflector with (a) $\gamma =60^{\circ }$, (b) $\gamma =90.01^{\circ }$: (1) $kc_{1}=12$, kc=12.1; (2) $kc_{1}=18$, kc=18.1.

If the internal termination of our rigid cavity moves to its open end, we arrive at the sphere-conical reflector (see figure 1a,b). The curves in figure 13 show the far field scattered from the reflectors with different radii of an aperture and the opening angles. Considering the behaviour of the curves in this figure, we can conclude that the reflectors mentioned above focus the scattering field along the axis $\theta =180^{\circ }$. We also observe the side lobs in these figures, which can be explained as the mirror scattering from the conical surface and the radiation of the edge waves. By comparing the far-field patterns scattered from the same soft and rigid open-ended cavities that are shown in figures 7 and 13, it is found that their shapes are very close. However, the magnitudes of the radiated fields are different.

**Figure 14.**
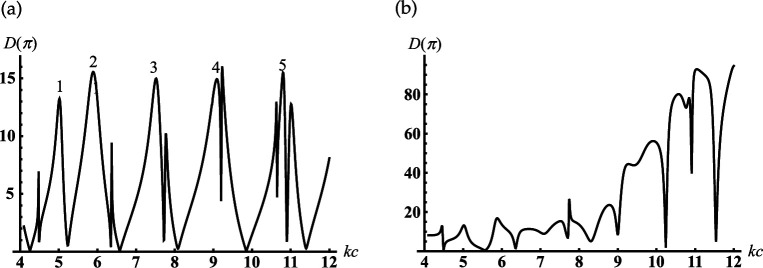
Dependencies of the backscattering on the termination coordinate kc for the rigid cone with $\gamma =150^{\circ }$: (a) $kc_{1}=4$; (b) $kc_{1}=kc-1$.

Consider the cone with γ>π/2. Under this condition, our scatterer becomes an open spherical resonator conjoined with the semi-infinite conical horn (see figure 1c). In figure 14a, we observe the dependence of the back-radiation from the open spherical resonator into the conical horn on the parameter kc with $kc_{1}=\mathrm{const}$. It is found from figure 14a that the peaks of the back radiation can be observed if the internal termination coordinate kc is located close to the resonant ones $k_{j}c$ for corresponding closed spherical and closed sphere-conical resonators. These peaks and the types of resonant vibration of the resonators mentioned above are shown in table 4. We see from this table that the joint spherical and sphere-conical modes with the index ν=1/2 (see peak numbers 1 and 5 in figure 14a) are excited in our resonator and radiated into the conical horn. Due to the end of the conical horn penetrating the spherical resonator ($c>c_{1}$), the resonant modes with the indexes $\nu_{2}$ and $\nu_{3}$ of the sphere-conical resonator are also radiated into the conical horn (see peak numbers 2–4 in figure 14a). In figure 14b, we represent the similar dependence if the depth of the horn end penetrating into the sphere is fixed, $k(c-c_{1})=1$.

**Table 4.** Dimensionless resonant radii of the closed rigid spherical vibrations $U_{\nu_{n},0,j}$ for $\gamma =150^{\circ }$ ; $\nu_{1}=1/2$ , $\nu_{2}=1.61565$ , $\nu_{3}=2.77797$ ; $k_{j}c$ is the position of the peaks in figure 14a.

peak number in figure 14a	1	2	3	4	5
type of vibration $U_{\nu ,0,j}$	$U_{\nu_{1},0,1}$	$U_{\nu_{2},0,2}$	$U_{\nu_{3},0,2}$	$U_{\nu_{2},0,3}$	$U_{\nu_{1},0,3}$
roots of equation $J_{\nu_{n}}(x)-2xJ_{\nu_{n}}^{\prime }(x)=0$	4.4934	6.10027	7.65414	9.37179	10.9041
$k_{j}c$	5.019	5.895	7.518	9.092	10.804

Therefore, the rigid sphere-conical scatterer, similar to that we observe for the soft one, supports the resonant vibration of two perfect closed resonators: spherical and sphere-conical. This phenomenon shows the possibility of tuning resonant excitation modes.

## 10. Conclusion

In this work, we have solved two new canonical wave diffraction problems of axially symmetric plane wave diffraction from the truncated cone with an internal spherical-cap termination. We consider the soft and the rigid cases. The problems are solved rigorously using analytical regularization techniques. The field is expressed through the conical and spherical eigenfunctions. The mode-matching technique is applied to derive the functional series equations for the rigid and soft problems. The infinite system of linear algebraic equation (5.11) is obtained from the functional series. This system has the same presentation for two of our cases. It is shown that the matrix operators of the obtained system of equations allow for selecting the singular parts and constructing the corresponding inverse operators in an analytical form for the soft and the rigid scatterers. These operators are applied to develop the analytical regularization technique and reduce the problems to the infinite systems of linear algebraic equations of the second kind, which remains uniformly valid for arbitrary cavity dimensions. Equations (6.12) and (6.16) are the key ones. Two types of such regularizations are considered: left- and right-hand side ones. The proposed technique is generalized by constructing the sets of regularizing operators for solving these problems. The resonance excitation of open-ended soft and rigid sphere-conical cavities are analysed analytically for the small size aperture of truncation. The new approximate expressions for determining the shifts of the resonant frequencies of closed sphere-conical resonators caused by the small size truncation of the conical vertex are obtained. Based on the numerical calculations, the resonant and scattering characteristics of the sphere-conical reflectors, spherical resonators conjoined with the semi-infinite conical horn and plane surface with the subsurface semi-spherical cavities are obtained. We studied the rigid and soft scatterers, calculating diffracted fields for various geometrical parameters and frequencies. We found that when the cavities are irradiated axial symmetrically by a plane acoustic wave from an open end, the diffracted fields are focused in the opposite direction; the frequency dependencies of backscattering have an oscillatory form, which depends on the truncation radius of the cavity, the dimension of the corresponding closed one and the type of the scatterer’s surface. Comparing the far-field patterns of the soft and rigid scatterers reveals that their shapes are similar but have different intensities. We found the low-frequency resonant excitation of the piston mode in a whole rigid dynamic system caused by the presence of an open-ended cavity.

## Data Availability

This article has no additional data.

## Appendix A. Transformation of Legendre series to Dirichlet series

The trivial solution (5.6) of equation (5.5) if J→∞ directly follows from the completeness of the Legendre functions $\{P_{\eta_{j}-1/2}(\pm \cos\;\theta ){\}}_{j=1}^{\infty }$, where $\eta_{j}$ is the positive roots of the transcendental equations (3.3). It is shown in [42] that the completeness of the Legendre functions $\{P_{\lambda_{n}}(x){\}}_{n=1}^{\infty }$ with fraction indices $\lambda_{n}\geq 1/2$ is a consequence of the completeness of $\{\cos(\lambda_{n}+1/2)x{\}}_{n=1}^{\infty }$. In order to prove this for our case, let us consider the Mehler–Dirichlet integral representation of the Legendre functions [35] as

(A 1a)Pηj−1/2(cos⁡θ)=2π∫0θcos⁡(ηjφ)cos⁡φ−cos⁡θdφ,if θ∈[0,γ),(A 1b)Pηj−1/2(−cos⁡θ)=2π∫θπcos⁡(ηj(π−φ))cos⁡θ−cos⁡φdφ,if θ∈(γ,π].

Suppose the series (5.5) is uniformly convergent in the determined angle regions. Let us substitute the expression (A 1) into equation (5.5) and change the summation and integration order. Then, we arrive at the homogeneous Abel equation, which allows only the trivial solution. This transforms the Legendre series (5.5) into the Dirichlet one as

(A 2)
$$\begin{array}{lcr} & \lim_{J\to \infty }\sum_{j=1}^{J}[\alpha_{j}e^{\eta_{j}z}+\beta_{j}e^{-\eta_{j}z}]=0, & \end{array}$$

where z=iθ if θ∈[0,γ) and z=i(π−θ) if θ∈(γ,π]; $\alpha_{j}=\beta_{j}=\Lambda_{j}/2$ are known linear forms. Equation (A 2) is formed by the series of exponents $\left\{e^{\pm \eta_{j}z}\right\}$, where $\eta_{j}$ is the zeros of the even entire functions of the exponential type, whose indicatrix of growth is determined as

h(φ)=limρ→∞⁡ln⁡|R(ρeiφ)|ρ=σ|sin⁡φ|.

Here, R(ν) is any of the Legendre functions from (3.3) that are considered as a function of the complex index $\nu =\rho e^{i\varphi }$; σ is the type of the Legendre function as an entire and even function of an argument ν. Then, as follows from the properties of the series of exponents [43] if the series (A 2) is uniformly convergent in the strip |Imz|≤σ, the homogeneous series equation (5.5) allows for only the trivial solution $\Lambda_{j}\to 0$ in the areas mentioned above for all j=1,2,3,..., when J→∞.

In order to determine the parameter σ for the Legendre functions, let us represent them in the form of the infinite Weierstrass product

(A 3)
$$\begin{array}{lcr} & R(\nu )=R(0)\prod_{n=1}^{\infty }\left(1-\frac{\nu^{2}}{\eta_{n}^{2}}\right). & \end{array}$$

Taking into account that the indicatrix of growth of the entire functions represented by the product (A 3) is determined as $\sigma =\pi \lim_{n\to \infty }(n/\eta_{n})$ [44] and using the asymptotic expressions for the roots of equations (3.3) as

(A 4a)ηn=νn=π(n−1/4)/γ+O(1/n),ηn=μn=π(n−1/4)/(π−γ)+O(1/n), D-problem, (A 4b)ηn=νn=π(n−3/4)/γ+O(1/n),ηn=μn=π(n−3/4)/(π−γ)+O(1/n), N-problem, 

we find that σ=γ if θ∈[0,γ) and σ=π−γ if θ∈(γ,π]. These estimations are correct for any of the D- or N-problems. Therefore, equations (A 2) and (5.5) allow only for the trivial solutions for all j=1,2,3,... if J→∞.
